# Enhanced tumor control and survival in preclinical models with adoptive cell therapy preceded by low-dose radiotherapy

**DOI:** 10.3389/fonc.2024.1407143

**Published:** 2024-10-09

**Authors:** Nahum Puebla-Osorio, Natalie Wall Fowlkes, Hampartsoum B. Barsoumian, Kristina Xega, Gitika Srivastava, Claudia Kettlun-Leyton, Sara Nizzero, Tiffany Voss, Thomas S. Riad, Christina Wong, Ailing Huang, Yun Hu, Joylise Mitchell, Mingee Kim, Zahid Rafiq, Kewen He, Duygu Sezen, Ethan Hsu, Fatemeh Masrorpour, Aurian Maleki, Carola Leuschner, Maria Angelica Cortez, Philipp Oertle, Marko Loparic, Marija Plodinec, Janet L. Markman, James W. Welsh

**Affiliations:** ^1^ Department of Radiation Oncology—Research, The University of Texas MD Anderson Cancer Center, Houston, TX, United States; ^2^ Department of Veterinary Medicine and Surgery, The University of Texas MD Anderson Cancer Center, Houston, TX, United States; ^3^ Takeda Development Centers Americas, Inc, Lexington, MA, United States; ^4^ ARTIDIS AG, Basel, Switzerland; ^5^ Medical College of Wisconsin, Milwaukee, WI, United States

**Keywords:** NSG, NOD-SCID-IL2R gamma mice, LDRT, low-dose radiotherapy, RT, radiotherapy, CAR-T cells, chimeric antigen receptor T cells, solid tumors

## Abstract

**Introduction:**

Effective infiltration of chimeric antigen receptor T (CAR-T) cells into solid tumors is critical for achieving a robust antitumor response and improving therapeutic outcomes. While CAR-T cell therapies have succeeded in hematologic malignancies, their efficacy in solid tumors remains limited due to poor tumor penetration and an immunosuppressive tumor microenvironment. This study aimed to evaluate the potential of low-dose radiotherapy (LDRT) administered before T-cell therapy to enhance the antitumor effect by promoting CAR-T cell infiltration. We hypothesized that combining LDRT with T-cell therapy would improve tumor control and survival compared to either treatment alone.

**Methods:**

We investigated this hypothesis using two NSG mouse models bearing GSU or CAPAN-2 solid tumors. The mice were treated with engineered CAR-T cells targeting guanyl cyclase-C (GCC) or mesothelin as monotherapy or in combination with LDRT. Additionally, we extended this approach to a C57BL/6 mouse model implanted with MC38-gp100+ cells, followed by adoptive transfer of pmel+ T cells before and after LDRT. Tumor growth and survival outcomes were monitored in all models. Furthermore, we employed atomic force microscopy (AFM) in a small cohort to assess the effects of radiotherapy on tumor stiffness and plasticity, exploring the role of tumor nanomechanics as a potential biomarker for treatment efficacy.

**Results:**

Our results demonstrated enhanced tumor control and prolonged survival in mice treated with LDRT followed by T-cell therapy across all models. The combination of LDRT with CAR-T or pmel+ T-cell therapy led to superior tumor suppression and survival compared to monotherapy, highlighting the synergistic impact of the combined approach. Additionally, AFM analysis revealed significant changes in tumor stiffness and plasticity in response to LDRT, suggesting that the nanomechanical properties of the tumor may be predictive of therapeutic response.

**Discussion:**

The findings of this study highlight the transformative potential of incorporating LDRT as a precursor to adoptive T-cell therapy in solid tumors. By promoting CAR-T and pmel+ T-cell infiltration into the tumor microenvironment, LDRT enhanced tumor control and improved survival outcomes, offering a promising strategy to overcome the challenges associated with CAR-T therapy in solid tumors. Additionally, the changes in tumor nanomechanics observed through AFM suggest that tumor stiffness and plasticity could be biomarkers for predicting treatment outcomes. These results support further investigation into the clinical application of this combined approach to improve the efficacy of cell-based therapies in patients with solid tumors.

## Graphical Abstract

Low-dose radiotherapy enhances the infiltration of adoptively transferred CAR-T cells to enhance tumor control and survival in solid tumors.

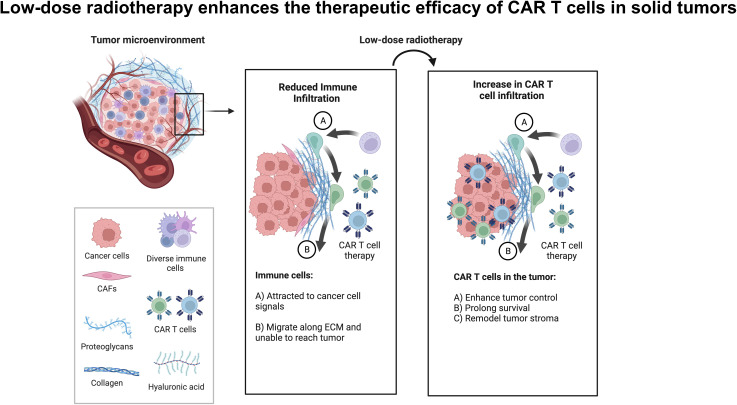

## Introduction

Chimeric antigen receptor T cell (CAR-T) therapy has been less effective in treating solid tumors compared to hematologic malignancies, partly attributed to the immunosuppressive signals within the tumor microenvironment (TME). Components like stroma, cytokines, chemokines, checkpoint proteins, and metabolites hinder the function and persistence of adoptively transferred immune cells (1–3). Tumor-associated macrophages, regulatory T cells (Tregs), and myeloid-derived suppressor cells (MDSC) release cancer-associated cytokines, such as TGF-β and IL-10, further suppressing immune responses (4–7). Collagen overproduction by cancer-associated fibroblasts (CAFs) also forms a rigid stromal barrier around tumors, shielding them from immune surveillance (8–11) Different radiotherapy approaches have been explored to address TME challenges. Hypofractionated radiotherapy (HFRT) applied to the primary tumor induces an abscopal effect in distant metastasis (12) HFRT applied to the primary tumor followed by low-dose radiotherapy (LDRT) to the secondary lesion has shown promise in reducing TGF-β and enhancing intratumoral immune cell infiltration (6, 13, 14). However, the ideal doses of non-ablative radiotherapy to achieve the maximum therapeutic response, particularly when preceding cell therapy, still need to be determined (14–16). Studies have shown radiation therapy (RT) can synergize with checkpoint inhibitors by modulating the stroma to enhance T-cell infiltration (13, 17–23). Our team discovered that applying LDRT directly to tumors modulates the tumor stroma and microenvironment without causing damage to normal tissues (6, 13). De Selm et al. found that a single dose of 2 Gy sensitizes tumor cells to immune rejection by CAR-T cells. The study, conducted in a pancreatic adenocarcinoma model, demonstrated that antigen-positive and antigen-negative tumor cells become susceptible to CAR therapy when exposed to this radiation dose. Their findings provide promising insights into the successful application of CAR therapy for heterogeneous solid tumors, especially when coupled with local radiation as a conditioning regimen, a common component of standard tumor care (24).

LDRT demonstrates significant potential in augmenting the effectiveness of adoptive CAR-T cell therapy against solid tumors by reshaping the TME and bolstering immune cell infiltration. Our study employs a novel strategy using T-cell therapy for solid tumors post LDRT, capitalizing on its beneficial impact on the TME. Through this integrated radioimmunotherapy approach, we aim to redefine the efficacy of cancer treatment.

We evaluated the impact of administering LDRT before T-cell therapy on tumor growth control and survival across three mouse models. NSG mice were implanted with GSU or CAPAN-2 cell lines, followed by LDRT before CAR-T cell infusion. Similarly, C57BL/6 mice were injected with MC38-gp100+ murine colon adenocarcinoma cells and treated with pmel+ T cells before or after LDRT administration. Our results demonstrate that the sequential application of LDRT before T-cell therapy significantly improves tumor control and extends survival compared to control groups across all experimental models.

Also, we evaluated the efficacy of tumor growth control in a bilateral tumor model, where only the primary tumor received LDRT before CAR-T cell infusion. Remarkably, tumors subjected to LDRT before CAR-T cell infusion exhibited significantly superior tumor control compared to unirradiated tumors within the same mouse.

Although we refer to the administration of LDRT with CAR-T cells as combined therapy throughout this manuscript, LDRT was consistently administered before CAR-T cell infusion in all our experiments to mitigate potential harm to the infused cells. The only exception was observed in the pmel experiment, where one group received adoptively transferred gp100-sensitized pmel cells before LDRT, as previously described.

In summary, our innovative approach of integrating LDRT before T-cell therapy demonstrates promising results in enhancing the infiltration of adoptively transferred T cells and improving treatment outcomes for patients with solid tumors while minimizing additional toxicity.

## Materials and methods

### Mouse models

We used 8–12-week-old NSG (NOD-SCID IL2Rgamma) male mice, and 8–12-week-old C57BL/6 mice purchased from the Department of Experimental Radiation Oncology at The University of Texas MD Anderson Cancer Center; all mice were housed in the Experimental Radiation Oncology animal facility at MDA. All mouse studies included a minimum of 5 mice per group and were conducted under guidelines from the Institutional Animal Care and Use Committee. The NSG mice were implanted subcutaneously in the right hind leg (0.5 x 10^6^ cells/mouse) with either GSU cells (Creative Bioarray cat #CSC-C6317J) or CAPAN-2 (2 x 10^6^ cells/mouse) cells (American Type Culture Collection, HTB-80). To create a bilateral tumor model in NSG mice, we first implanted the primary tumor on the right hind leg (0.5 x 10^6^ cells/mouse) on day zero, followed by a secondary implantation on the left hind leg (0.5 x 10^6^ cells/mouse) five days later. The C57BL/6 mice were implanted similarly with (0.5 x 10^6^ cells/mouse) MC38 murine colon adenocarcinoma cells expressing mouse gp100 [a gift from Dr. Patrick Hwu]. Procedures for the LDRT and T cell therapies are described below.

### Radiotherapy

Radiation was delivered at total doses of 2 Gy, 4 Gy, 12 Gy, 24 Gy, or 36 Gy as follows: 1 fraction of 2 Gy; 1 fraction of 4 Gy; 2 fractions of 1 Gy each; 3 fractions of 4 Gy each; 3 fractions of 8 Gy each; or 3 fractions of 12 Gy each. We also used 4 fractions of 1Gy in later studies, and for the pmel study, we used 4 fractions of 1.4 Gy (total 5.6 Gy). The latter dose was chosen from a clinical radiation dose delivered to patients with metastatic disease (25). Radiation was delivered to the implanted tumors with a custom-built Cs-137 unit, with the rest of the mouse body shielded to avoid off-target effects.

### *In vitro* irradiation of naïve T cells

We exposed freshly collected and sorted human CD3+ naive T cells to two fractions of 1Gy radiation in triplicate, with a 24-hour interval between exposures, *in vitro* using the X-RAD320 irradiator (Precision X-Ray Irradiation; Madison, CT, USA.) After exposure, we collected cells at 24, 48, and 72 hours to extract RNA for Nanostring analysis. We utilized the nSolver software to analyze the irradiation-exposed cells compared to their controls. Using the normalized dataset, we performed fold induction analysis to assess the impact of irradiation exposure on various genes, including those involved in TCR signaling and DNA repair pathways.

### Adoptive cell therapy

For adoptive cell therapy, we used second-generation CAR-T cells against GSU cells expressing guanyl cyclase-C (GCC) or against CAPAN-2 cells expressing mesothelin as follows: A leading signal domain was linked to the codon-optimized single heavy and light chain fragments recognizing GCC or mesothelin, followed by a spacer tethered to the transmembrane domain of CD28 (anti-GCC CAR) or CD8 (anti-mesothelin CAR). The construct was finalized with the intracellular domains of CD28 (anti-GCC CAR) -or 4-1BB (anti-mesothelin CAR)- and CD3. Mice were administered intravenously with 1×10^6^, or 2.5 × 10^6^ anti-GCC CAR-T cells (for the GSU model) or 1×10^5^ anti-mesothelin CAR-T cells (for the CAPAN-2 model) 24h after the last dose of radiation. As a control, untransduced (UTD) T cells from the same donor were administered. Cells were maintained in liquid nitrogen until the day of the infusion when they were thawed at 37°C, their viability was determined, and 100 μL aliquots in PBS were prepared for injection. For the third mouse model, 5x10^6^ pmel^+^ T cells were adoptively transferred into C57BL/6 mice implanted with gp100^+^ MC38 cells at 24 h after the final dose of RT.

### Tumor control and mouse survival

After implantation, tumors were left to grow to ~7 mm in diameter (~170 mm^3^), which was reached by days 8 or 10 depending on the tumor model utilized; this size was ideal for testing various LDRT doses and schedules. Tumors were measured with high-precision calipers twice weekly, and the volumes calculated from tumor length and width measurements as described by Tomayco and Reynolds (26). Mouse survival was recorded over the experimental periods, and the survival curves were calculated with the Kaplan-Meier method (GraphPad). Tumor specimens were obtained when they reached the permissible size or at the end of the experiment; after volumes were measured, tumors were divided in half for assessment of tumor-infiltrating lymphocytes and creation of tissue microarrays (TMAs) as described below.

### Statistical analysis

The differences in tumor growth among groups in each experiment were calculated using one-way ANOVA and Tukey’s multiple comparison tests (p< 0.05). In addition, to factor in the unexpected loss of tumor measurements due to the tumor burden and death of affected mice, we implemented a one-way ANOVA mixed-effects analysis and Tukey’s multiple comparison tests (p-value < 0.05). The differences in survival among groups were calculated using Chi-square analysis and Log-rank (Mantel-Cox) test for curve survival comparison (p<0.05).

### Isolation and flow cytometry of tumor-infiltrating lymphocytes

Half of each tumor specimen was subjected to single-cell dissociation followed by gradient centrifugation with Lymphoprep (1.077 g/mL, StemCell Technologies). Blood samples were collected via tail vein in live animals and by heart puncture in sacrificed animals and preserved in heparin (10U/mL blood). The isolated cells were stained for flow cytometry with the following antibodies, all from BioLegend: anti human CD4 (PE/Dazzle-594; cat #357412, clone A161A1), CD8 (PerCP/Cyanine 5.5; cat #344710, clone SK1), CD45 (Brilliant Violet 650; cat #304044, clone HI30), CD3 (Brilliant Violet 711; cat #317328, clone OKT3), CD69 (allophycocyanin [APC]; cat #310910, clone FN50), CD279 (PD1) (PE/Cyanine7; cat #3621616, clone A17188B), CD197 (CCR7) (Brilliant Violet 605; cat #353224, clone G043H7), CD45RA (Brilliant Violet 785; cat #304140, clone HI100), and anti-mouse CD16/32 (cat #101302, clone 93). Doublets were removed by plotting forward-scatter height (FSC-H) vs. forward-scatter area (FSC-A); live single-cell populations were selected by plotting side scatter-area (SSC-A) vs. a Live/Dead marker, followed by identification of the lymphocyte population. CD4^+^ and CD8^+^ T cell subpopulations were then identified from the total CD3^+^ fraction. Tumor-infiltrating lymphocytes were subjected to flow cytometry with a Cytek Aurora cytometer. The gating strategy is described in online **Supplementary Figure 1**. In addition, when required, we generated t-SNE maps to display T-cell populations using Cytofkit (27), an R-based interface available in Bioconductor (https://www.bioconductor.org/packages/3.5/bioc/html/cytofkit.html).

### Tissue processing and immunostaining

Portions of tumor specimens were fixed in 10% neutral buffered formalin followed by paraffin-embedding, tissue sectioning (4 μm), and staining with hematoxylin and eosin (H&E). The H&E-stained slides were inspected visually and two 3-mm cores were selected from each tumor block for inclusion in a TMA. The TMA block was sliced in 4-μm sections and stained with a Leica Bond RX auto stainer and Opal 7-color multiplex kit (Akoya Biosciences) to detect human CD4, CD8, PD1, Ki67, granzyme B, and PanCK, with DAPI used for counterstaining. Multiplex IF-stained slides were imaged with a Leica Versa 8 fluorescent digital slide scanner. Slides were viewed by using Imagescope and HALO software. Biomarkers were quantified with a Leica cellular immunofluorescence algorithm. Quantitative data were exported in Excel and graphed and analyzed with GraphPad Prism version 9. One-way analysis of variance was used for statistical analyses.

### Tissue nanomechanics

The nanomechanical characterization utilizing atomic force microscopy (AFM) was conducted using the ARTIDIS research device, which is an advanced AFM-based technique that enables precise and detailed characterization of the mechanical properties of biological samples at the nanoscale, facilitating a deeper understanding of cellular and tissue mechanics in various biomedical applications. Biopsies obtained from mice tumors were securely mounted on standard TPP 9.2 cm^2^ dishes from TPP (Switzerland) using a 2-component epoxy resin glue. We prepared 2-5 samples for measurements from mice receiving LDRT only, CAR-T only group, or LDRT before CAR-T cell therapy. The ARTIDIS chip holder pre-equipped with triangular DNP-S10 D probes from Bruker AFM Probes (USA) was utilized for nanomechanical measurements. These probes possess specific properties, including a nominal spring constant of 0.06 N m−1, a cantilever length of 205 µm, a tip height of 6 µm, and a tip radius of 20 nm. The spring constant in the air was determined through a fully automated calibration process employing the thermal tune method described by Sader et al. (28) Deflection sensitivity calibration was performed by acquiring force curves at the bottom of TPP 9.2 cm^2^ dishes filled with degassed and sterile Custodiol solution. The ARTIDIS device integrated optical microscopes from Leica (Germany) and Navitar (USA), allowing for imaging and precise positioning control of both the cantilever and the sample.

To determine the elastic modulus of the samples, the AFM was operated in force-volume mode. This involved capturing 20 x 20 force-displacement curves arranged in square arrays (force maps), each map covering an area of 20 x 20 µm^2^. This process resulted in 400 force curves per map. We acquired force maps for each sample at a minimum of 20 equidistant spots across the sample. During the acquisition of force-displacement curves, indentations ranging between 0.2 and 3 µm were sampled, applying a defined maximum force load (29, 30) of 1.8 nN and an indentation speed of 16 µm s−1.

Subsequently, the AFM data were analyzed using the ARTIDIS analysis software. The recorded curves were transformed into force vs. tip-sample distance curves. For each sample, all the force maps were evaluated to assess the quality of the force curves. We excluded maps with quality rates below 75% from further analysis. We considered signal-to-noise ratio, baseline tilt, and curve twist factors in selecting suitable maps for analysis (31). We evaluated 17 nanomechanical parameters between mice treated with radiation only, CAR-T cells only, and those treated with LDRT plus CAR-T cell therapy, with varying cell quantities (1, 2.5, and 5 million cells per mouse). The backward elastic modulus was determined using the contact area and slope, employing the Oliver and Pharr model (32), as previously established in the literature (30, 33). The elastic modulus of each force curve was spatially plotted in 2D to generate a stiffness map for the different samples. A Gaussian distribution was derived from the collected stiffness values across the sample groups. The force maps were further processed for visualization purposes using Gwyddion software (34).

## Results

### Determining radiotherapy dose for use with cell therapy

**Figure 1:**
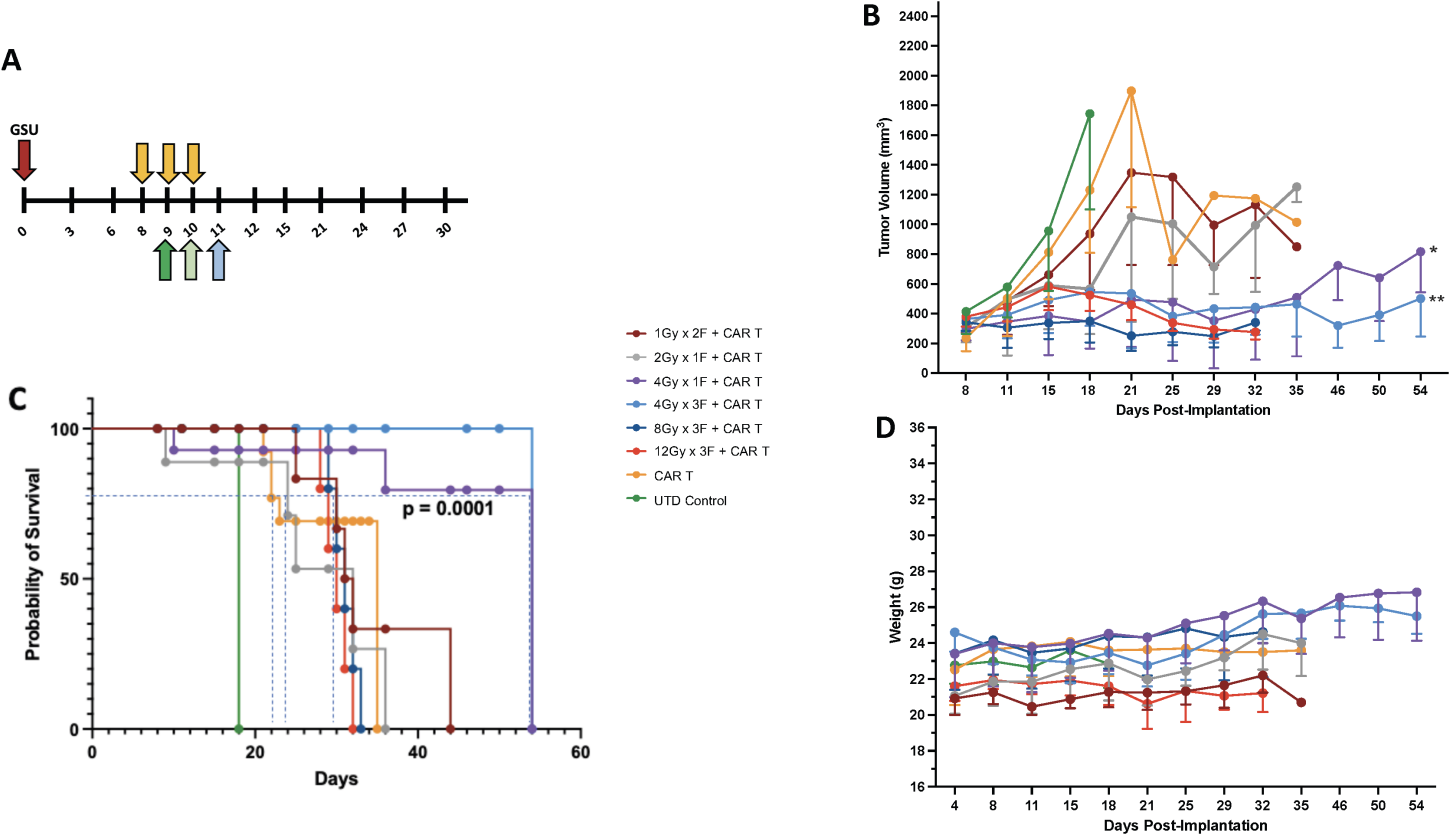
Establishing the best radiation dose and schedule for combining with CAR-T cell therapy. **(A)** We used the GSU tumor model to determine the proper radiation dosage combined with cell therapy; cell therapy was administered 24h after the last fraction of radiotherapy (each radiation fraction is indicated by a yellow arrow). If radiation was given in one fraction on day 8 (2 Gy or 4Gy), the infusion was administered on day 9 (dark green); if the radiation included two doses (1Gy in 2 fractions) on days 8 and 9, the mice received the infusion 24h after the last fraction on day 10 (light green). If the mice received 3 fractions on days 8, 9, and 10 (4, 8, or 12Gy in 3 fractions), the T cell infusion was administered 24h after the last fraction on day 11 (light blue). Two regimens (1 fraction of 4 Gy [purple lines] or 3 fractions of 4 Gy each [light blue lines]) in combination with CAR-T cell therapy led to superior tumor control **(B)** and **(C)** survival and **(D)** had little to no effect on body weight. Compared to the CAR-T-only group, the groups that received 4Gy x 1F (purple) or 4Gy x 3F (light blue) showed statistically better tumor control (*p<0.004 [4Gy x 1F + CAR-T cells vs. CAR-T cells] and **p<0.001 [4Gy x 3F+CAR-T cells vs. CAR-T cells]; one-way ANOVA and Tukey’s multiple comparison tests; n=5.), and survival (*** p<0.0001, [4Gyx 1 fraction + CAR-T cells or 4Gy x 3 fractions + CAR-T cells vs. CAR-Ts or the remaining combinations]; Long-rank Mantel-Cox test). The two highest-dose schedules [3 fractions of 8 Gy each (dark blue) and 3 fractions of 12 Gy each (44, 45)] led to skin ulceration at the irradiation site.

To effectively control tumor growth, adoptively transferred T cells must reach and infiltrate beyond the tumor stroma. Multiple factors affect this event, including the tumor microenvironment, its localization, and growth status at the time of treatment. Our study aimed to determine the best radiation dose to enhance tumor control before CAR-T cell therapy. Using an experimental model with human gastric cancer in NSG mice, we simplified the process by implanting tumors only in one leg. This allowed for targeted radiation and more straightforward tumor measurement. We found that injecting 1 million GSU cells per mouse created tumors reaching a suitable size (~170 mm^3^) within eight days, ideal for testing different radiation doses and schedules (online **Supplementary Figure S2A**). This model setup enabled us to explore the optimal radiation approach for enhancing CAR-T cell effectiveness against tumor growth. For the GSU experiments, mice from each experimental group received focal irradiation on day 8 post-implantation, followed by the infusion of a single intravenous dose of 2 x 10^6^ CAR-T cells recognizing guanyl cyclase-C (GCC) expressed on the GSU tumor cells 24 h after receiving their last dose of radiotherapy (**Figure 1A**). Mice receiving untransduced (UTD) naïve T cells or CAR-T cells alone succumbed to the tumor burden by day 18 and 35 after implantation, respectively (**Figures 1B, C**). Also, mice receiving 1Gy x 2F or 2 Gy x 1F prior to CAR-T cells had a marginal effect in controlling tumor growth, succumbing to the tumor burden on day 35 (**Figures 1B, C**). Although mice that received 12Gy x 3F or 8Gy x 3F followed by CAR-T cell therapy had an efficient control of tumor growth (**Figure 1B**); such regimens led to skin ulceration at the irradiation site that prompted early euthanasia on day 32. Notably, LDRT doses of 12 Gy (4-Gy x 3 fractions) or 4 Gy (4Gy x 1 fraction) administered before anti-GCC CAR-T cells, controlled tumor growth (**Figure 1B**) without adverse reactions at the irradiation site and prolonged survival until the termination of the experiment on day 54 after implantation (**Figure 1C**) (*** p<0.0001, [4Gy x 1 fraction+ CAR-T cells or 4Gy x 3 fractions+ CAR-T cells vs. CAR-T cells or the remaining combinations]; Long-rank Mantel-Cox test). Moreover, twice-weekly measurements of body weight revealed no weight loss related to CAR-T-cell infusion in any group (**Figure 1D**). To minimize potential RT-related side effects, we opted to use 4Gy x 1 fraction or 1Gy x 4 fractions in subsequent experiments, in part because the latter regime resembles the dose and schedule used to treat metastatic disease in the clinic (1.4Gy x 4 fractions) (25). Further testing is warranted to explore the potential therapeutic effects of administering three fractions of 4Gy (totaling 12 Gy) before CAR-T cell therapy. This intervention could regulate tumor growth and enhance survival in different mouse tumor models. In summary, combining CAR-T cell therapy with LDRT rather than high-dose radiotherapy makes therapeutic sense due to LDRT’s low side effects, and its role in modulating the tumor stroma, as demonstrated by our recent findings (13, 25).

### Radiotherapy optimizes the use of cell therapy

**Figure 2:**
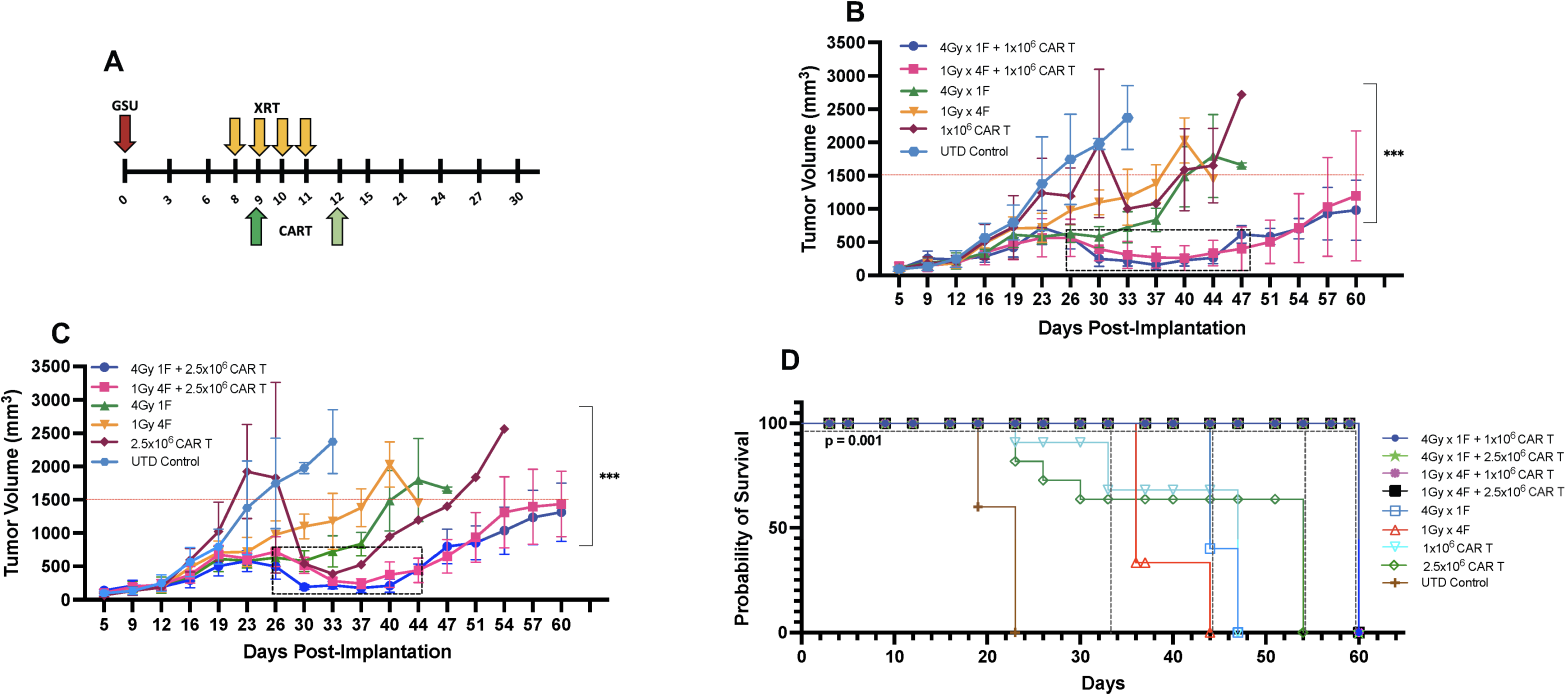
Determining the ideal CAR-T cell dose combined with radiation for mice bearing subcutaneous GSU gastric carcinoma tumors. **(A)** In an efficacy study using the GSU tumor model, if the radiation was given in one fraction (each radiation fraction is indicated by a yellow arrow) on day 8 (4Gy) post-tumor implantation, the infusion was administered on day 9 (dark green); if the radiation included four doses (1Gy in 4 fractions) on days 8, 9, 10, and 11, the mice received the infusion 24h after the last fraction on day 12 (light green). In a single experiment, we included two doses of CAR-T cells combined with two dose regimens of radiotherapy. **(B, C)** Mice receiving 1×10^6^ anti-GCC CAR-T cells combined with either 1Gy x 4 fractions or 4Gy x 1 fraction showed superior tumor control (B; days 26–47) relative to the mice given the same radiation doses with 2.5 × 10^6^ cells (C; days 26–44). **(D)** Mice that received dual therapies (4Gy x 1 fraction, 1Gy x 4 fractions plus 1 or 2.5 × 10^6^ CAR-T cells; black, pink, green, and blue) showed extended survival relative to the other experimental treatments (p<0.001, [4Gy x 1 fraction, 1Gy x 4 fractions plus 1 or 2.5 × 10^6^s CAR-T cells vs. monotherapies]; Long-rank Mantel-Cox test). (n= 5 mice per group).

LDRT enhances the anti-tumor immune response (13, 25) and is gaining traction as an important approach to improve clinical outcomes. Recent data show that efficacy from autologous CAR-T cells or tumor-infiltrating lymphocyte therapies (35) depend on the fitness of the T cells used for their manufacturing or expansion; this fitness is inherent to each patient and is an absolute determinant of desirable therapeutic outcomes (36, 37). Because CAR-T cells alone often have high anti-tumor efficacy in xenograft models, we intentionally selected CAR-T cell resistant tumor models and/or suboptimal CAR-T numbers that have low to medium efficacy as a monotherapy to assess the extent of RT-induced enhancement in tumor control and survival. To determine the optimal number of CAR-T cells to be used with our chosen LDRT schedules (1Gy x 4 fractions or 4Gy x 1 fraction), we followed the schema depicted in **Figure 2A**. In this study, we conducted experiments using NSG mice implanted with GSU cells. On day 8, RT was administered, followed by the infusion of different doses of anti-GCC CAR-T cells (1x10^6^ or 2.5 × 10^6^) 24 hours after the final LDRT dose (day 9 or day 12; **Figure 2A**, indicated by dark green or light green arrows).

Our findings demonstrated that combining LDRT with any of the CAR-T cell doses resulted in significantly better tumor control than CAR-T cells alone, untreated T cells, or four fractions of 1Gy radiation therapy alone (**Figures 2B, C**).

Notably, 4Gy x 1 fraction LDRT followed by 1x10^6^ CAR-T cells showed significantly superior tumor control compared to all CAR-T doses alone (4Gy x 1 fraction + 1x10^6^ CART vs. 1x10^6^, or 2.5 × 10^6^ CAR-T alone; p<0.0001, and 0.0004; n=5 mice per group). Moreover, the use of 4Gy x 1 fraction LDRT preceding any CAR-T dose (1x10^6^, or 2.5 × 10^6^) was significantly more effective than using 1Gy x 4 fractions LDRT followed by 2.5 × 10^6^ CAR-T cells (4Gy x 1 fraction + 1x10^6^ CAR-T vs. 1Gy x 4 fractions + 2.5 × 10^6^ CAR-T; p<0.002; n=5 mice per group).

In addition to tumor control, the combination treatments led to 100% survival up to day 60 (**Figure 2D**). We observed a period of robust tumor control from day 26 to day 47 (21 days) in mice receiving 1x10^6^ CAR-T cells after the administration of 1Gy x 4 fractions or 4Gy x 1 fraction and from day 26 to day 44 (18 days) in mice receiving 2.5 × 10^6^ CAR-T cells after 1Gy x 4 fractions LDRT (**Figures 2B, C**, indicated by the dotted box).

Comparatively, 100% of mice receiving UTD succumbed to the tumor burden by day 23, and mice receiving CAR-T cells alone died by day 47 (1x10^6^ CAR-T cells) and 54 (2.5 × 10^6^ CAR-T cells) (**Figure 2D**). Mice treated with LDRT only (1Gy x 4 fractions or 4Gy x 1 fraction) also exhibited no control of tumor growth and succumbed to the tumor burden at day 44 and 47, respectively) (**Figure 2D**). Interestingly, one mouse that received 1Gy x 4 fractions before 1x10^6^ CAR-T cells demonstrated tumor growth suppression throughout the experiment (online **Supplementary Figure 3D**). Detailed tumor records for individual mice can be found in online **Supplementary Figures 3A–I**.

Based on these findings, LDRT can potentially enhance the efficacy of anti-GCC CAR-T cell therapy in this experimental model.

**Figure 3:**
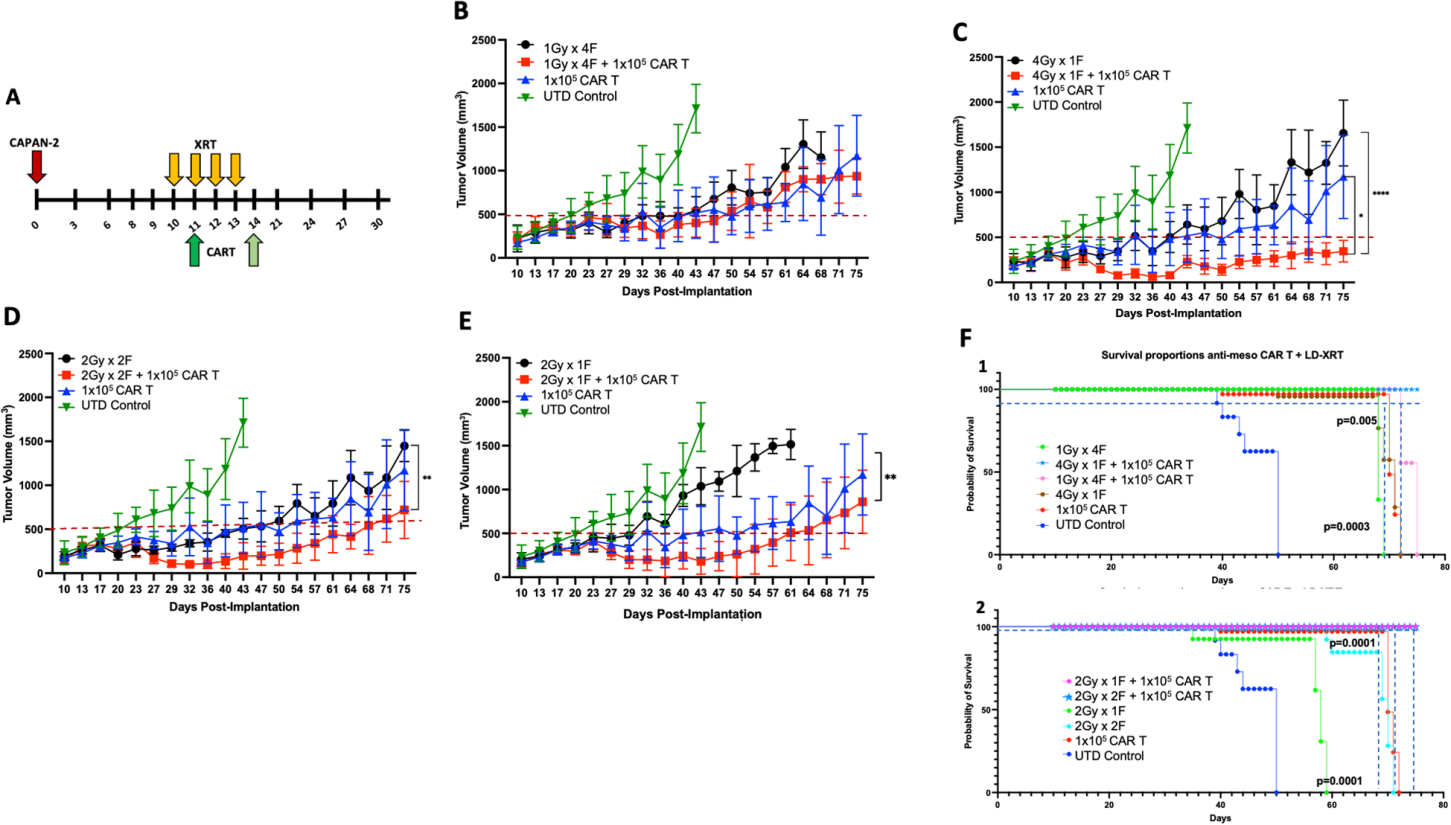
CAR-T cell therapy combined with radiotherapy in mice bearing subcutaneous CAPAN-2 tumors. **(A)** In the efficacy studies using the CAPAN-2 tumor model the radiation was given in one fraction on day 10 (4Gy) post-tumor implantation when tumors reached ~7mm in diameter and the infusion was administered on day 11 (dark green); if the radiation included four doses (1Gy x 4 fractions) on days 10, 11, 12, and 13; the mice received the infusion 24h after the last fraction on day 14 (light green). In a single experiment, we determined the efficacy of multiple radiation doses combined with a single dose of CAR-T cell therapy. In all four radiation dose groups in this experiment **(B–E)**, mice treated with dual therapy showed superior tumor control compared with mice receiving monotherapies. Mice treated with 1Gy x 4 fractions plus CAR-T cell therapy showed marginally better control of tumor growth relative to radiation or CAR-T cells alone **(B)**. Notably, the use of 4Gy x 1 fraction combined with CAR-T cell therapy resulted in the most significant tumor control **(C)** and a 100% survival rate **(F1)** at day 75 [i.e., compared to mice receiving CAR-T cells (p<0.005) or 4Gy x 1 fraction of LDRT (p<0.0003)]. Also, relative to LDRT alone (p<0.0001) and CAR-T therapy alone (p<0.0001), 2 Gy x 2 fractions combined with anti-mesothelin CAR-T cells also significantly improved tumor control **(D)** and survival **(F2)**. In addition, 2 Gy x 1 fraction + CAR-T cells **(E)** was marginally, but not significantly better than 1Gy x 4 fractions + CAR-T cells. (n=5 mice per group).

Next, to assess whether CAR-T cell therapy after LDRT was also effective in a different tumor model, we implemented a similar therapeutic approach in NSG mice implanted with the mesothelin-positive human CAPAN-2 cell line. We first determined that by subcutaneously implanting 2x10^6^ CAPAN-2 cells per mouse, the tumor grew to an optimal size for radiotherapy of ~7 mm in diameter on day 10 post-implantation (online **Supplementary Figure S2A**). The CAPAN-2 cell line grew slower than the GSU cell line *in vivo* (online **Supplementary Figure S2A**). Because the CAPAN-2 cells were previously determined to be more sensitive to CAR-T treatment than the GSU model, we tested fewer CAR-T cells per mouse than in the GSU experiments, i.e., 0.5×10^5^, 1×10^5^, or 2×10^5^. We found that 0.5×10^5^ or 2×10^5^ anti-mesothelin CAR-T cells led to low or maximum antitumor response, respectively, (online **Supplementary Figure S2B**), and 1×10^5^ CAR-T cells per mouse had intermediate killing efficacy. The use of the latter dose made therapeutic sense as we sought to assess whether a moderate number of CAR-T cells combined with LDRT enhanced the overall response. We implanted all grouped NSG mice with 2×10^6^ CAPAN-2 cells in the right hind leg and recorded the tumor growth twice weekly. The mice-bearing tumors were irradiated with the following doses of radiotherapy: 1Gy x 4 fractions, 4Gy x 1 fraction, 2 Gy x 1 fraction, and 2 Gy x 2 fractions. All radiotherapy doses preceded the infusion of 1×10^5^ anti-mesothelin CAR-T cells, in which the CAR-T cells were administered 24 hours after the final radiation dose according to **Figure 3A**.

The results indicate that neither the groups treated with CAR-T cell nor radiotherapy alone controlled tumor growth as effectively as the combined therapies. In the CAPAN-2 tumor model, treatment with 1Gy x 4 fractions before CAR-T cell therapy showed marginal enhancement of control of tumor growth relative to CAR-T cells or radiation alone, although 60% of these mice survived 75 days after implantation (**Figures 3B, F1**). Notably, the use of 4Gy x 1 fraction prior anti-mesothelin CAR-T cell therapy led to the most effective tumor control (**Figure 3C**) and a 100% survival rate (**Figure 3F1**) at day 75 [i.e., size of tumors ~ 350 mm^3^ after 4Gy x 1 fraction + CAR-T vs. ~ 1150 mm^3^ after CAR-T only (p*<*0.005) or ~1650 mm^3^ after 4Gy x 1 fraction only (p*<*0.0003)]. Also, 2 Gy x 2 fractions combined with anti-mesothelin CAR-T cells also significantly improved tumor control (**Figure 3D**) and survival (**Figure 3F2**) relative to LDRT alone [*p<*0.0001]) and CAR-T therapy alone (p<0.0001). In addition, 2 Gy x 1 fraction + CAR-T cells (**Figure 3E**; values for individual tumor records are shown in online **Supplementary Figure S4**) was marginally, but not significantly better than 1Gy x 4 fractions + CAR-T cells (tumor volumes of ~800 mm^3^ vs. ~900 mm^3^ at Day 75). These findings confirmed that LDRT plus anti-mesothelin CAR-T cell therapy was effective for controlling tumor growth and extending survival in NSG mice.

To test whether radiotherapy combined with cell therapy leads to similar outcomes in non-immunosuppressed mice, we used LDRT with adoptive cell transfer (ACT) of pmel-sensitized T cells in two separate pilot experiments. Before their infusion, the pmel splenocytes were activated with anti CD3/CD28 beads and maintained in IL-2 and IL-15- supplemented culture for 96 h. We implanted 5x10^5^ MC38-gp100^+^ tumor cells on the right hind leg of C57BL/6mice and allowed the tumors to develop until they reached ~7 mm diameter before irradiating them with 1.4Gy x 4 fractions (Day 9 post-implantation). To test whether radiotherapy after the cell infusion affected the outcome, we adoptively transferred each mouse with 5x10^6^ pmel-sensitized T cells 24 h before initiating radiotherapy (Day 8 post-implantation). Also, we adoptively transferred pmel T cells 24 h after the last administered radiotherapy fraction (Day 13 post-implantation) (online **Supplementary Figure S5A**). Our results indicate that mice receiving pmel-sensitized T cells 24 h before LDRT or 24h after the last LDRT dose showed better tumor control than mice receiving cell therapy only (day 30 post-tumor implantation, ACT 24h before LDRT vs. ACT, p=0.0003; ACT 24h after LDRT vs. ACT, p=0.0001) (Online **Supplementary Figures S5B, C**). These results are in line with the findings in the tumor xenograft models described here. Thus, combining the adoptive cell transfer of effector T cells with radiotherapy results in better tumor control.

### Low-dose radiotherapy enhances the infiltration of adoptively transferred T cells

The efficacy of anti-tumor cell therapy requires robust intratumoral T-cell infiltration and sustained and active effector function. However, tumors generate multiple barriers within the TME that impair the infiltration of T cells. Tumor-infiltrating T cells suffer numerous changes affecting their anti-tumor activity. Using radiotherapy in low doses helps modulate the TME by increasing the infiltration of infused anti-tumor T cells and promoting beneficial changes, including the reduction of T-regs and TGF-β, and enhancing the proliferation of M1 macrophages, among others (13, 25).

**Figure 4:**
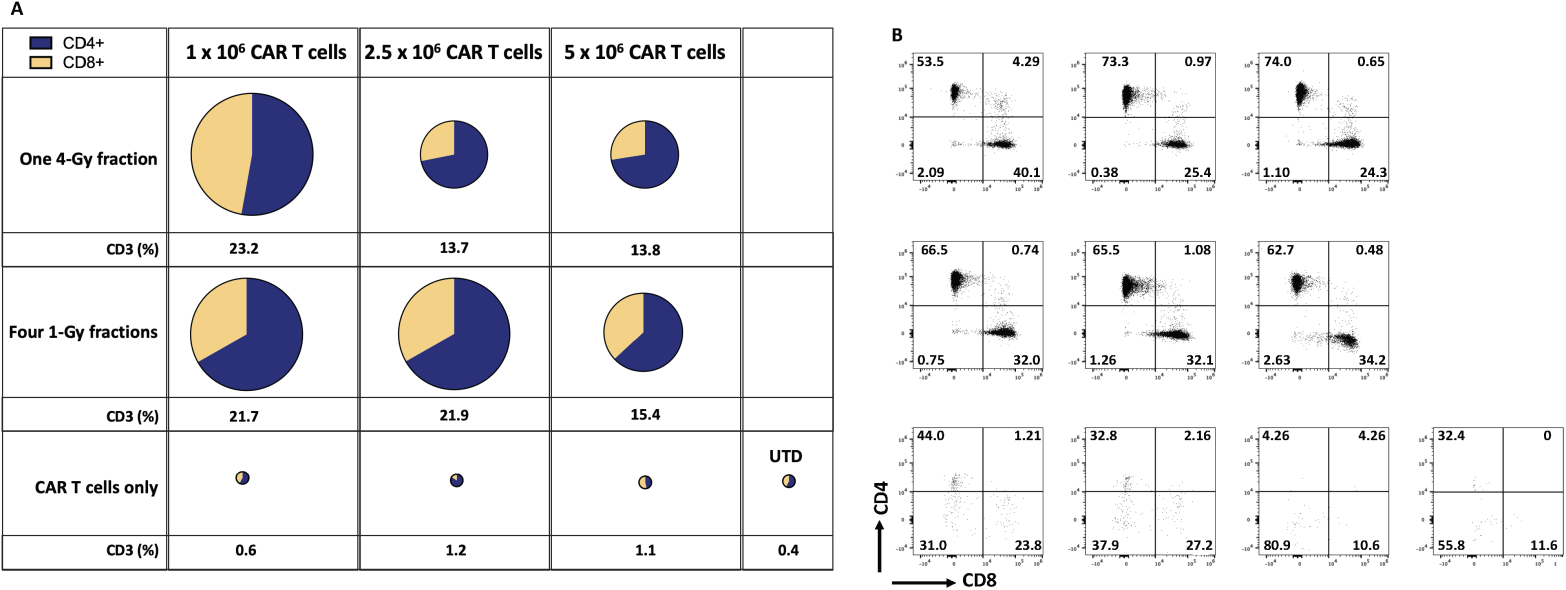
Radiation therapy enhances the infiltration of adoptively transferred anti-GCC CAR-T cells into the GSU gastric carcinoma tumor microenvironment. The average (n=3) of absolute numbers of CD3^+^ CAR-T cells were determined in tumor samples from the indicated experimental groups. **(A)** The relative sizes of the pies represent the amount of CD3^+^ cells, and the two colors indicate the relative proportions of CD4^+^ cells (blue) and CD8^+^ cells (yellow). Mice receiving dual therapy (4Gy x 1 or 1Gy x 4 fractions with 1×10^6^, or 2.5 × 10^6^ CAR-T cells) showed significant increases in the intratumoral infiltration of CAR-T cells relative to mice receiving CAR-T cells only (4Gy x 1 fraction + 1x10^6^ CAR-T vs, 1 and 2.5 × 10^6^ CAR-T cells. p<0.01, 0.004, and 0.04, respectively), and 1Gy x 4 fractions ^+^ 10^6^ CAR-T cells showed a higher percentage of CD3^+^ cells than 1 and 2.5 x 10^6^ CAR-T cells alone (1Gy x 4 fractions + 1x10^6^ CAR-T vs, 1, 2.5 x 10^6^ CAR-T cells; p<0.02, 0.01, respectively.) **(B)** Starting from the upper leftmost sample, examples of CD4 and CD8 distributions by flow cytometry, corresponding to the same treatment group as in panel **(A)**; samples from mice receiving CAR-T or UTD cells only showed a smaller number of tumor-infiltrating T cells. Tumor-infiltrating T cells were collected when mice reached the maximum permissible tumor volume and succumbed to the tumor burden (mostly in mice receiving monotherapy). However, mice that received the dual therapy were terminated at day 60, and samples collected. The gating strategy is described in online **Supplementary Figure 1**. (n=5 mice per group).

Indeed, our results in the current study imply that LDRT enhanced the infiltration of infused CAR-T cells in the GSU model. To assess the persistence of infused anti-GCC CAR-T cells, we measured their presence in mice’s bloodstream and tumor microenvironment (TME). We collected blood samples at two points: upon death from tumor burden (typically occurring earlier in mice receiving only CAR-T cells or untransduced donor T cells [UTD cells]) and at the experiment’s end for mice that survived after combination therapy with CAR-T cells and LDRT. Thus, samples were collected at different time points for each group: on days 20-33 for the UTD mice; days 24-37 for the 5×10^6^ CAR-T cell group; days 24-44 for the 1×10^6^ CAR-T cell group; or days 24-54 for the 2.5 × 10^6^ CAR-T cell group. We also collected samples at the end of the experiment (on day 60) from mice receiving dual therapy with 1Gy x 4 fractions or 4Gy x 1 fraction and infused with 1×10^6^, 2.5 × 10^6^, or 5×10^6^ CAR-T cells. Mice receiving the dual therapy (4Gy x 1 or 1Gy x 4 fractions with 1×10^6^, 2.5 × 10^6^, or 5×10^6^ CAR-T cells) showed significant increases in the intratumoral infiltration of CAR-T cells (human CD3^+^ cells; **Figures 4A, B**; **Supplementary Figures S6A**, **S7**) relative to mice receiving anti-GCC CAR-T cells only (4Gy x 1 fraction + 1x10^6^ CAR-T cells vs 1, and 2.5 CAR-T cells. p<0.01, 0.004, and 0.04, respectively), and 1Gy x 4 fractions + 1x10^6^ CAR-T cells showed higher percentage of CD3^+^ cells than 1 and 2.5 × 10^6^ CAR-T cells alone (1Gy x 4 fractions + 1x10^6^ CAR-T vs 1, 2.5 × 10^6^ CAR-T cells; p<0.02, 0.01, respectively.) Thus, regardless of the number of cells infused, the intratumoral infiltration of adoptively transferred CAR-T cells was consistently higher in mice receiving dual therapy. (**Figure 4**; **Supplementary Figures S6A**, **S7**).

To summarize, we found that infusing the fewest CAR-T cells (1x10^6^ per mouse) after LDRT (4Gy x 1 or 1Gy x 4 fractions) led to better tumor control, extended survival, and better infiltration of CAR-T cells into tumors than using 2.5 × 10^6^ CAR-T cells after LDRT. To confirm our findings, we repeated this experiment by using 4Gy x 1 or 1Gy x 4 fractions combined with 1×10^6^ anti-GCC CAR-T cells and measured chimeric human CD3^+^ cells in the blood. Our findings validated the previous experiment showing that the dual therapy led to better tumor control and better intratumor infiltration of the infused CAR-T cells. Specifically, administering 1Gy x 4 fractions or 4Gy x 1 fraction combined with 1×10^6^ CAR-T cells led to higher percentages of chimeric human CD3^+^ T cells in the blood (analyzed on day 60) than groups treated with UTD cells or CAR-T cells only analyzed on day 30 in the blood (endpoint; online **Supplementary Figures 6**, **7**). Further, the administration of dual therapy (1Gy x 4 or 4Gy x 1 before 1×10^6^ anti-GCC CAR-T cells resulted in higher percentages of tumor-infiltrating CAR-T cells than receipt of UTD cells only or CAR-T cells only. Notably, these results also show that although CD4^+^ cells dominate the CD4^+^/CD8^+^ cell ratio in the blood and the tumor, the percentage of CD8^+^ T cells in the tumor increases, which indicates a potential expansion of these cells due to their engagement with the targeted tumor proteins.

**Figure 5:**
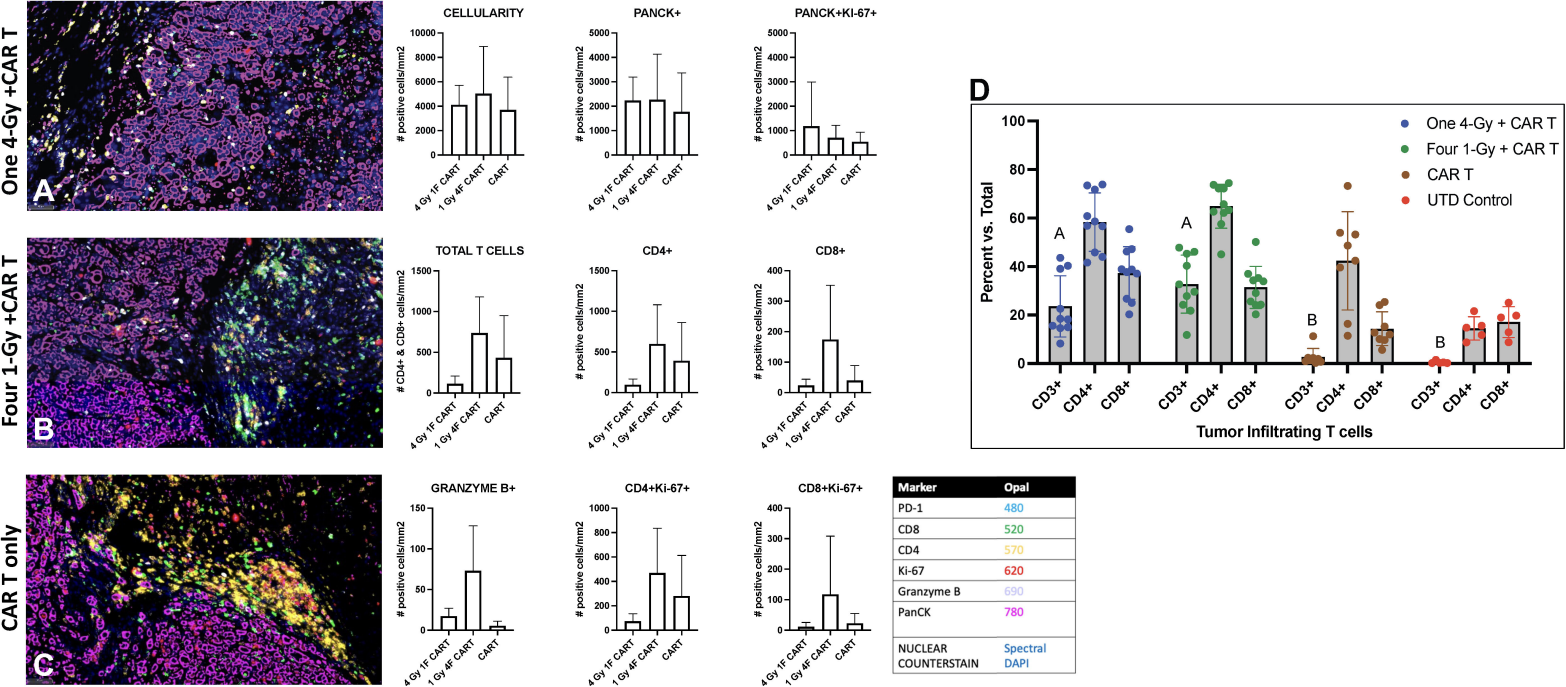
Radiation therapy enhances the infiltration of adoptively transferred anti-GCC CAR-T cells into GSU gastric carcinoma tumors in NSG mice. Mice given 1Gy x 4 fractions each **(B)** showed enhanced infiltration of CAR-T cells (total T cell, CD4^+^, and CD8^+^), increased proliferation (Ki67^+^) of CD4 and CD8 cells, and increased numbers of granzyme B^+^ cells in the tumor microenvironment relative to mice receiving 4Gy x 1 fraction **(A)** or CAR-T cells only **(C)**. Images captured at 20x. (n=5 mice per group). **(D)** Quantitative analysis of tumor-infiltrating human CD3+ T cells shows that mice treated with a combination of radiotherapy either one 4-Gy fraction or four 1-Gy fractions, and CAR-T therapy (n=10) exhibit a significantly higher percent of tumor-infiltrating T cells compared to those treated with CAR-T therapy alone or untreated (UTD) control mice (columns with different letter are statistically different, p-value < 0.0001, mixed-effect model analysis with multiple comparisons).

We also assessed the intratumoral infiltration of CAR-T cells by using multiplex immunohistochemical staining in a cross-section of the GSU tumors used for flow cytometry analyses described above (**Figure 5**). In that experiment, mice receiving 1Gy x 4 fractions followed by CAR-T cells showed higher intratumoral infiltration of CAR-T cells and increased CD4^+^ and CD8^+^ subpopulations relative to mice receiving 4Gy x 1 fraction prior CAR-T cells or CAR-T cells only, as well as an increase in the proliferation (Ki67^+^) of CD4^+^ and CD8^+^ T cells and an increase in granzyme B production (**Figures 5A–C**). Moreover, treatment with CAR-T cells only led to more CD4^+^ cells being retained outside the tumor (**Figure 5C**) than treatment with combination therapy. In short, even though statistical significance was not reached (probably because of the heterogeneity and low numbers of evaluated samples), administering 1Gy x 4 fractions seems to favor the increased intratumoral infiltration of total CAR-T cells and the effector molecule granzyme B, as well as increasing the CD4^+^ and CD8^+^ T cells relative to CAR-T cells only or 4Gy x 1 fraction followed by CAR-T cell therapy (**Figure 5**). Also, we performed quantitative analysis of tumor-infiltrating human CD3+ T cells by flow cytometry, showing that mice treated with both radiotherapy (one 4-Gy or four 1-Gy fractions) and CAR-T therapy (n=10) have a significantly higher percentage of tumor-infiltrating T cells compared to those treated with CAR-T therapy alone or untreated (UTD) control mice (p-value < 0.0001, mixed-effect analysis with multiple comparisons) (**Figure 5D**).

We repeated the above experiments with the CAPAN-2 model. The CAPAN-2-tumor-bearing mice showed comparable levels of infiltration of the anti-mesothelin CAR-T cells among all treatment groups: percentages of CD3^+^ cells were 43% for CAR-T cells only; 45% for CAR-T cells + 2 Gy x 1 fraction; 48% for CAR-T cells + 2 Gy x 2 fractions; 38% for CAR-T cells + 1Gy x 4 fractions; and 32% for CAR-T cells + 4Gy x 1 fraction (online **Supplementary Figure 8**). The t-SNE maps [Cytofkit (27)] show the distribution of CD4 (cluster 10, green) and CD8 cells (cluster 7, yellow.) Although treatment with CAR-T cells + 4Gy x 1 fraction had relatively fewer tumor-infiltrating CAR-T cells (38%), those mice also had the best tumor control and survival compared with the other groups (**Figure 3C**).

**Figure 6:**
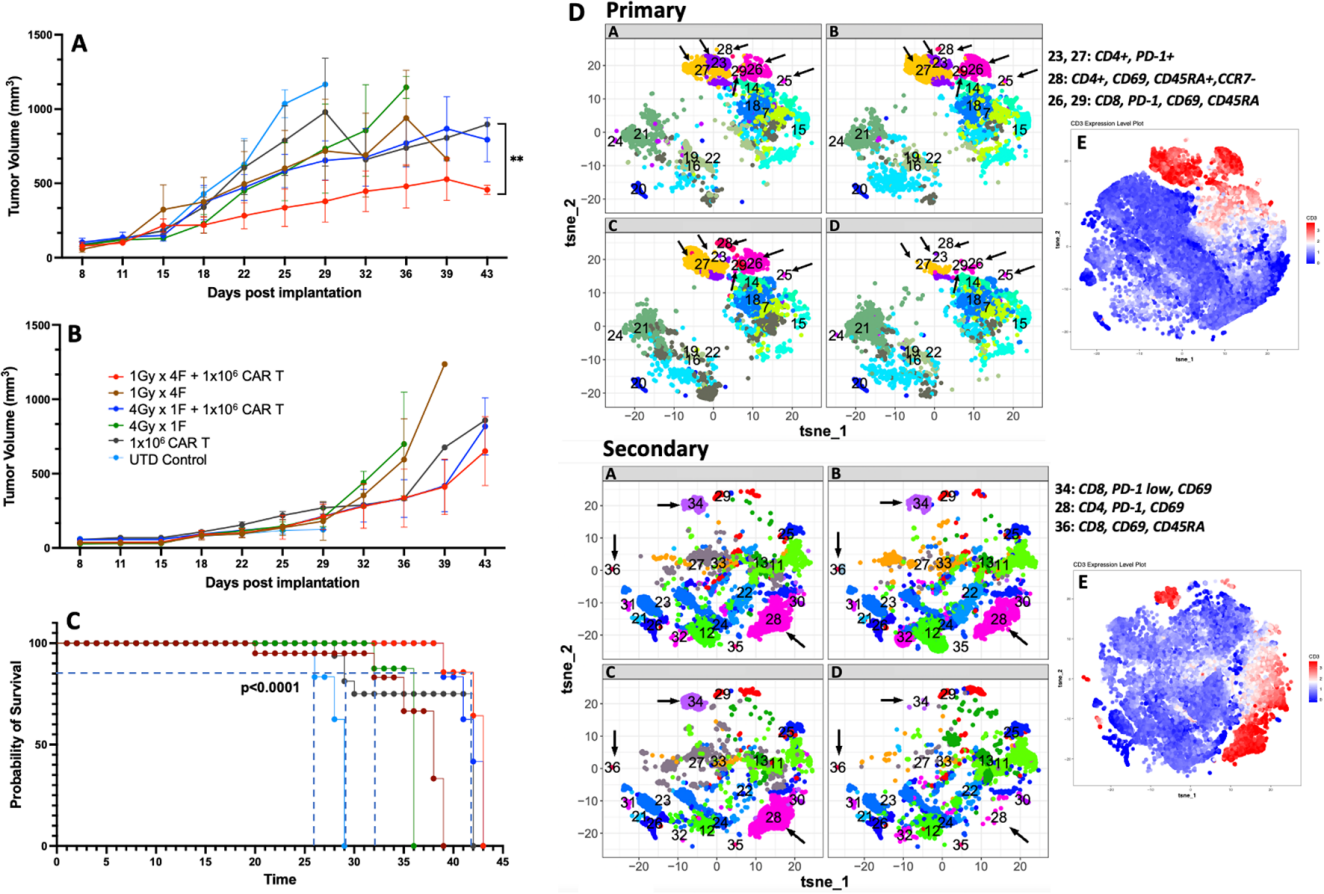
1Gy x 4 fractions (1Gy x 4F) or 4Gy x 1 fraction (4Gy x 1F) combined with anti-GCC CAR-T cell therapy exhibited significantly better tumor growth control and survival than CAR-T therapy alone in a bilateral tumor model. **(A)** Primary tumor growth comparison shows a significant difference (p<0.02) between mice receiving 1Gy x 4 fractions vs. mice receiving CAR-T cells only. Four mice from the CAR-T group were euthanized by day 30 due to tumor burden and only one mouse survived to the end of the experiment. Also, mice receiving 1Gy x 4 fractions combined with CAR-T cells showed significantly better control in the primary tumor (p<0.009) than 4Gy x 1 fraction + CAR-T cell therapy. Although no significant differences were observed in the control of the secondary tumor **(B)**, the dual therapy 1Gy x 4 fractions combined with CAR-T cell therapy showed enhanced control of the primary tumor and a significant survival rate (**(C)**, p<0.0001) than mice receiving CAR-T cell therapy or radiation therapy alone. **(D)** We also analyzed the CAR-T phenotype on cells collected at the time of their death (n=3). With exception to the UTD control, all groups had representative populations in the primary tumors including CD4^+^PD-1^+^ (C23), terminally differentiated CD4^+^ T cells (CD4^+^CD69^+^, CD45RA^+^, CCR7^-^; C28), and activated CD8^+^cells, (CD69^+^, PD-1^+^; C26). Also, in the secondary tumors, except for the UTD group, we did not observe changes in the expression of CD4 (C28) or CD8 (C34, C36) between the groups. E) The red clusters in both primary and secondary tumors, represent the distribution of the CD3^+^ populations. (n= 5 mice per group). The t-SNE maps were generated using the R-based software interface Cytofkit (https://www.bioconductor.org/packages/3.5/bioc/html/cytofkit.html).

Next, we used a bilateral tumor model to understand whether the dual therapy was sufficient to control tumor growth in non-irradiated tumors. Our results showed that using 1Gy x 4 fractions or 4Gy x 1 fraction before anti-GCC CAR-T cell therapy exhibited significantly better control of tumor growth in the primary tumor than mice receiving CAR-T cell therapy alone (p<0.02, **Figure 6A**). Also, mice receiving 1Gy x 4 fractions combined with CAR-T cells showed significantly better control in the primary tumor (p<0.009) than 4Gy x 1 fraction plus CAR-T cell therapy (**Figure 6A**). As expected, mice that received the dual therapies showed significantly better survival (p<0.0001) than mice receiving CAR-T cell therapy or radiotherapy alone (**Figure 6C**). All mice (n=5) receiving UTDs succumbed to the tumor burden by day 29. Four mice from the CAR-T group were eliminated by day 30 due to tumor burden, and only one mouse survived to day 39. We observed no significant differences in the control of the secondary non-irradiated tumor (**Figure 6B**) across treatment groups. Next, we analyzed the phenotype of CAR-T cells collected from primary and secondary tumors at the endpoint (**Figure 6**, primary and secondary; online **Supplementary Figure 9** shows the corresponding heatmaps to the t-SNE maps generated by Cytofkit (27).) Except for the untreated (UTD) control group, all groups exhibited representative populations in the primary tumors, including CD4^+^PD-1^+^ (cluster -C23), terminally differentiated CD4^+^ T cells (CD4^+^CD69^+^CD45RA^+^CCR7-; C28), and activated CD8^+^ cells (CD69^+^PD-1^+^; C26). Furthermore, in the secondary tumors, differences in the expression of CD4 (C28) and CD8 T cells (C34, C36) were not observed among all groups except for the UTD group.

The outcomes of this bilateral tumor experiment affirm the pivotal role of local low-dose radiotherapy in achieving enhanced tumor control when combined with CAR-T cell therapy. Notably, using LDRT proves a safe approach, as it does not compromise the populations of adoptively transferred CAR-T cells. We inferred this from the efficacy of combined therapy in controlling tumor growth, particularly in models where radiotherapy is not directly administered to adoptively transferred CAR-T cells. Nevertheless, in a smaller experiment, we aimed to determine the impact of applying LDRT directly to naïve T cells on their immune function and repair capabilities. Our investigation focused on assessing the effects of low-dose radiation therapy (LDRT) on human CD3+ naive T cells. Following LDRT exposure, we collected cells for RNA extraction and Nanostring analysis at specific times. Our analysis revealed differentially expressed genes associated with T cell receptor (TCR) signaling and DNA repair pathways through fold induction analysis. The gene expression data unveiled that LDRT induced moderate changes in pathways crucial for T cell function, with upregulation of genes such as TP53, COPS6, and FANCD2, indicating potential modulation of cell cycle progression and DNA repair mechanisms (online **Supplementary Figure 6B**). Interestingly, LDRT exposure also triggered the upregulation of genes within the PI3K/AKT/mTOR pathway, potentially promoting cell survival and proliferation. Additionally, modest upregulation of T cell activation and co-stimulation markers (CD3D, CD27, CD28) suggests partial T cell activation. The observed expression changes in immune checkpoint molecules (CTLA4, LAG3, TIGIT) and cytokines (IL2, IL10, IL4, TNF) underscore a dynamic immune response elicited by LDRT. Notably, the upregulation of CD8A, CD8B, and NFKB1 indicates enhanced cytotoxic activity in irradiated T cells (online **Supplementary Figure 6C**). While these findings offer an initial understanding of LDRT’s effects on normal donor non-activated T cells, further *in vivo* studies are imperative and currently underway to assess its impact on the function of adoptively transferred T cells. This study lays the groundwork for further exploration of LDRT’s influence on T cell-based cancer immunotherapies.

### A distinctive tissue nanomechanical signature in tumors treated with low-dose radiotherapy combined with CAR-T cells

**Figure 7:**
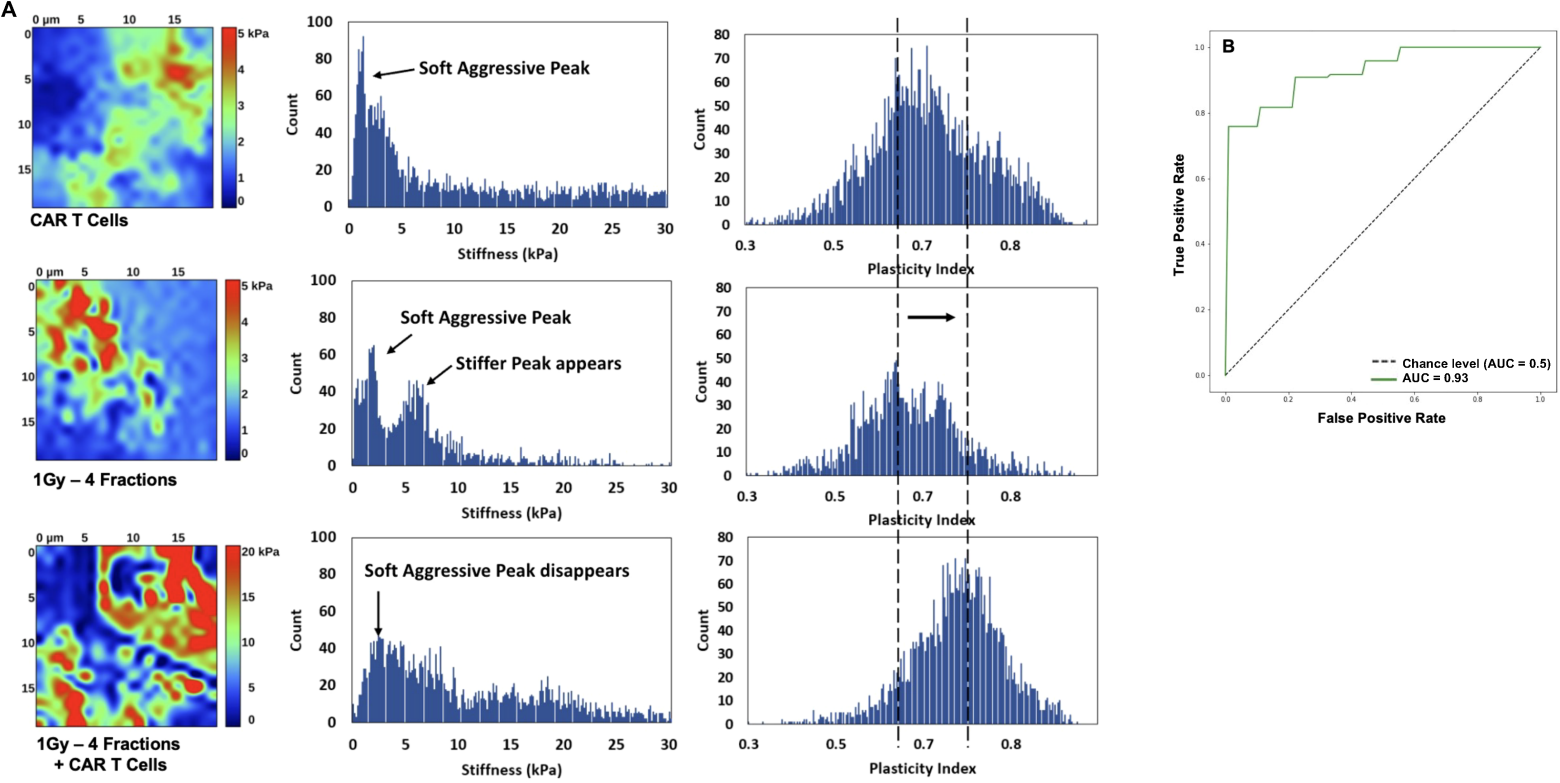
The distinctive signature of CAR-T cell therapy combined with LDRT is revealed through the combined analysis of plasticity index and stiffness. Mice receiving the combined therapy exhibited maps with varying stiffness and heightened plasticity index values and a reduction of aggressive soft cells. Additionally, there is an increase in stiffness of the second cellular peak and the emergence of a stiffer matrix peak around 10 kPa, resulting in an overall stiffening of the matrix **(A)**. Most samples exhibit a single peak in the plasticity domain, shifting towards higher plasticity with combined CAR-T cell and LDRT treatment. In contrast, mice treated solely with LDRT exhibit maps with heterogeneous softness and a low plasticity index. The ROC analysis yields significant results, with a 92% sensitivity, 100% specificity, and 95% accuracy, defining the tissue mechanical phenotype in the combined therapy as “PlasHi StiffHet.” In contrast, either monotherapy, RT, or CAR-T cells is categorized as “PlasLo StiffLo” **(B)**. Samples treated solely with low-dose radiation or CAR-T cells manifest a soft peak below 2 kPa, corresponding to aggressive cancer cells. Some samples also display a stiffer cell signature in the 2-5 kPa range. (n=5 mice per group).

To understand the effect of the combined low-dose radiotherapy (1Gy x 4 fractions) and CAR-T cell therapy on the stiffness and plasticity of the tumor stroma at the nanoscale level, we employed atomic force microscopy (AFM). Using AFM allowed us to quantitatively measure the mechanical properties of the cancer cells and obtain a unique nanomechanical signature associated with therapeutic efficacy (30, 31, 33). Our findings suggest that the combined use of the plasticity index and the stiffness or modulus backward approach, employed to derive mechanical properties like elastic modulus from force-distance curves acquired during indentation experiments, effectively delineates the impact of the combined therapy on the tumor stroma. This method reverses the indentation process to precisely ascertain the sample’s elastic properties, providing unique insights into the effects of the treatment. Samples from mice receiving CAR-T cell infusion combined with LDRT demonstrated heterogeneously stiffer maps with higher plasticity index values, which refers to the measure of the tissue’s ability to undergo plastic deformation when subjected to mechanical forces. In contrast, lower plasticity index values indicate that a tissue is more rigid and less likely to deform when subjected to mechanical forces (30, 31). In comparison, samples from mice treated solely with LDRT or CAR-T cell therapy exhibited heterogeneously softer maps with lower plasticity index values (**Figure 7A**). The receiving operating characteristic (ROC) (38) curve analysis resulted in a 92% sensitivity, 100% specificity, and an accuracy of 95% (**Figure 7B**), defining the tissue mechanical phenotype in the combined therapy as plasticity high/stiffness heterogeneous (PlasHi StiffHet). Conversely, the signature associated with LDRT treatment alone was labeled as plasticity low/stiffness low (PlasLo StiffLo.).

**Figure 7A** presents a detailed histogram illustrating stiffness and plasticity index measurements for representative samples. Samples treated solely with LDRT or CAR-T cells displayed a distinct soft peak below 2 kPa, indicative of soft and aggressive cancer cells. Additionally, three out of five samples in each treatment group exhibited a stiffer cell signature within the 2-5 kPa range.

These samples displayed a soft matrix, with maximum stiffness values ranging from 1.5 to 4 kPa. A noticeable shift in the signature was observed when comparing these measurements to those obtained from samples treated with LDRT before CAR-T cells. In samples subjected to the combined treatment, the soft peak diminished in four out of five samples and completely disappeared in one sample, suggesting a reduced presence of soft, aggressive cells. The second cellular peak exhibited increased stiffness and more significant heterogeneity, indicating a potential signature of activated CAR-T cells warranting further investigation. Furthermore, a stiffer matrix peak emerged around 10 kPa, accompanied by an overall stiffening of the matrix, extending to 30 kPa.

Most analyzed samples displayed a single heterogeneous peak in the plasticity domain, which notably shifted towards higher plasticity in samples treated with the combined therapy. Specifically, among samples treated solely with LDRT, all five displayed a dominant peak around 0.6-0.65, with two out of five samples also presenting a second peak around 0.8. In contrast, in samples treated with CAR-T cells after LDRT, four out of five exhibited a peak between 0.7 and 0.8, with two also displaying a peak around 0.6. These findings highlight the potential of the ARTIDIS signature as a biomarker for effective CAR-T cell therapy combined with LDRT in tumor tissues.

In summary, employing AFM to explore the nanoscale effects of LDRT preceding CAR-T cell therapy on tumor stroma, a distinctive tissue nanomechanical signature emerged. This signature, labeled “PlasHi StiffHet,” was characterized by heterogeneously stiffer maps with higher plasticity index values. In contrast, samples treated solely with LDRT or CAR-T cells exhibited a different signature labeled as “PlasLo StiffLo” with softer maps and lower plasticity index values. The analysis revealed a significant shift in the plasticity domain, suggesting that the identified ARTIDIS signature could be a biomarker for effective CAR-T cell infiltration in tumor tissues. The study showcased the potential of nanomechanical profiling to elucidate the mechanical phenotypes associated with therapeutic efficacy in combined cancer treatments.

## Discussion

Although immunodeficient mice are not a perfect model for testing the effectiveness of LDRT combined with cell therapy, they are nevertheless useful for testing therapeutic interventions against solid tumors. It is important to note that NSG models only partially translate into the clinic due to their limitations, such as lacking a functional immune system. However, despite these limitations, they remain valuable for preliminary testing, provide critical insights into therapeutic interventions, and help guide further research, making them an essential tool in the early stages of therapy development. We demonstrated that using LDRT combined with CAR-T cell therapy enhanced the control of tumor growth in NSG mice implanted with GSU or CAPAN-2 cells. Our approach included testing LDRT in several doses and schedules, delivered directly to implanted tumors, followed by a single intravenous injection of CAR-T cells recognizing either GCC or mesothelin 24 hours after the final LDRT fraction was delivered. Mice that received LDRT followed by CAR-T cell therapy survived longer and demonstrated significantly better tumor control compared to mice receiving monotherapy (i.e., CAR-T cells only, radiation LDRT only or untreated (UTD) T cells). We further chose to minimize the risk of RT-related side effects (e.g., skin ulceration) by focusing on a single dose of 4Gy or a low-dose of 1Gy x 4 fractions. We introduced using 4Gy divided into four fractions (1Gy x 4F) to emulate current dosing to treat metastatic disease in the clinic (25).

We also sought the ideal number of CAR-T cells for use as a single injection with the smallest possible effective LDRT dose. For the GSU model, we tested two doses of anti-GCC CAR-T cells (1×10^6^, 2.5 × 10^6^ cells/mouse) with two LDRT schedules (1Gy x 4 fractions and 4Gy x 1 fraction); for the CAPAN-2 model, we tested four LDRT schedules (1Gy x 4 fractions, 4Gy x 1 fraction, 2 Gy x 1 fraction, and 2 Gy x 2 fractions) and one dose of anti-mesothelin CAR-T cells (1×10^5^cells per mouse). In this model, combining anti-mesothelin CAR-T cells with 1Gy x 4 fractions or 4Gy x 1 fraction both significantly enhanced tumor control and prolonged survival for up to 75 days after tumor-cell implantation. Although the mice treated with CAR-T cells exhibited similar survival rates in the pancreatic model, their tumor control was less effective compared to the combination therapy. Additionally, we substantiated the efficacy of dual therapy in immunocompetent mice. In these experiments, C57BL/6 mice were implanted with MC38-gp100+ colon adenocarcinoma cells. We administered LDRT at 1Gy x 4 fractions and infused pmel-sensitized T cells, either before or after the radiation treatment. Our findings mimicked those in the immunodeficient NSG mouse models, in that mice treated with adoptively transferred pmel-sensitized T cells combined with LDRT enhanced the control of tumor growth as compared to mice treated with cell therapy alone. This effect is likely due to the role of LDRT in modulating both the tumor stroma and the tumor microenvironment as evidenced previously (6, 13). We used the pmel model to demonstrate that combining low-dose radiation therapy (LDRT) with cell therapy is more effective than cell therapy alone. Although we did not design the experiment to show survival, which is essential for understanding treatment impact, this was due to the lack of mouse-generated CAR-T cells to replicate our experiments using human CAR-T cells. Recognizing the need for complete characterization, including survival outcomes, we are now developing a humanized mouse model. This model will better emulate human CAR-T cell specificity and allow for a comprehensive evaluation of the combined therapy’s benefits.

Our findings unequivocally emphasize that the meticulous process of combining low-dose radiation (LDRT) with cell therapy demands a nuanced and tailored approach, eschewing a “one-size-fits-all” paradigm. The critical factor lies in carefully titrating doses of both radiation and CAR-T cells for optimal efficacy. For instance, administering 1Gy x 4 fractions combined with CAR-T cell therapy in the GSU model moderately controlled tumor growth and extended survival. However, this approach yielded different results in the CAPAN-2 model, where the most favorable response was observed with a single 4-Gy fraction coupled with 1 × 10^5^ anti-mesothelin CAR-T cells, maintaining tumor volumes below 500 mm³ and ensuring mouse survival for up to 75 days post-implantation.

It is crucial to stress that the choice of CAR-T cell dose is equally pivotal and must be carefully considered regarding both transduction efficiency and the cells’ effectiveness in killing targeted tumor cells. Invariably, compared with mice receiving CAR or untransduced T cells, those treated with combinations of CAR-T cells and LDRT exhibited similar levels of tumor infiltration by CAR-T cells, as assessed by total CD3+ cells gated on the SSC-A vs. FSC-A lymphocyte fraction.

Although tumor-infiltrating CAR-T cells were not collected simultaneously across treatment groups in the GSU tumor model, a time course study would accurately elucidate the extent of intratumor infiltration of CAR-T cells. Our results showed that mice receiving CAR-T cell monotherapy had significantly lower numbers of tumor-infiltrating CAR-T cells than those receiving dual therapy (CAR-T cells combined with RT), as assessed by gradient isolation of tumor-infiltrating T cells. This disparity is likely due to differences in survival, as mice receiving CAR-T cell therapy died at least a month earlier than those receiving the dual therapy.

Our results underscore the importance of personalized dosing strategies for radiation and CAR-T cell therapies to maximize therapeutic outcomes in different tumor models.

CD4 T cells are known to arrive earlier than CD8 T cells at the site of infection or tissue damage. This earlier arrival is crucial for orchestrating the subsequent immune response, as CD4 T cells help recruit and activate other immune cells, including CD8 T cells. This temporal sequence ensures a robust and coordinated immune attack against the pathogen or damaged tissue.

The results of our study align with this understanding. Our findings using multiplex immunohistochemical staining in the GSU model confirmed that mice treated with 1Gy x 4 fractions of radiotherapy showed an overall increase in total infiltrating T cells, including CD4+ and CD8+ T cells, compared to mice given only CAR-T cells or CAR-T cells with 4Gy x 1 fraction. Although these findings were not statistically significant, they are consistent with previous reports and clinical data indicating that LDRT (1.4Gy x 4 fractions) preferentially enhance the intratumor infiltration of CD4+ T and NK cells (6, 7, 13, 39). This observation reinforces the role of CD4 T cells in the early stages of immune infiltration and their potential impact on the overall immune response within the tumor microenvironment.

Moreover, by using a bilateral tumor model, we demonstrated that mice treated with either 4Gy x 1 fraction or 1Gy x 4 fractions with CAR-T cells led to significantly improved control of the primary (irradiated) tumor than mice with CAR-T cells alone. However, no significant differences in the control of the secondary tumor were observed. Our findings also revealed that the survival rate of mice treated with the combined therapy was significantly higher than those treated with either CAR-T or radiotherapy alone. This model emphasized the importance of administering low-dose radiotherapy locally, in conjunction with CAR-T cell therapy, to achieve superior tumor control. Also, our results indicate moderate changes induced by the direct exposure of normal donor non-activated T cells to two fractions of 1Gy in pathways crucial for T-cell function and the upregulation of genes promoting cell survival and proliferation. The observed upregulation of genes such as TP53, COPS6, and FANCD2 suggests potential modulation of cell cycle progression and DNA repair mechanisms in response to LDRT exposure, aligning with previous studies on the impact of radiation therapy on DNA damage repair pathways (40). Moreover, the modest upregulation of T cell activation and co-stimulation markers (CD3D, CD27, CD28), alongside dynamic changes in immune checkpoint molecules (CTLA4, LAG3, TIGIT) and cytokines (IL2, IL10, IL4, TNF), highlights the intricate interplay between LDRT and the immune response. Notably, the enhanced cytotoxic activity (CD8A, CD8B, and NFKB1) observed in irradiated T cells suggests a potential mechanism contributing to the improved tumor control observed in our study. These findings reinforce the importance of integrating low-dose radiotherapy with CAR-T cell therapy to optimize treatment outcomes.

We utilized atomic force microscopy (AFM) to investigate the impact of CAR-T cell therapy combined with LDRT on tumor stiffness and plasticity. Our findings reveal a distinctive characterization of the tumor stroma when subjected to the combined therapy. Mice receiving LDRT and CAR-T cell therapy exhibit stiffer maps and higher plasticity index values. Conversely, mice treated solely with LDRT display softer maps and lower plasticity index values.

The ROC analysis achieved notable results with a 92% sensitivity, 100 specificities, and accuracy of 95%, categorizing the mechanical phenotype of CAR-T cells as “PlasHi StiffHet.” Samples treated exclusively with LDRT or CAR-T cells exhibit a soft peak associated with aggressive cancer cells. In contrast, samples treated with the combined therapy show a reduced presence of these cells and increased stiffness. The plasticity peak shifts towards higher values in samples treated with CAR-T cells and RT, indicating effective tumor control.

Our results on the nanomechanical changes in the tumor stroma underscore the importance of mechano surveillance by immune cells, including T cells. This concept, which refers to the ability of immune cells to sense and respond to the mechanical properties of their environment (41–43), plays a crucial role in their activation, motility, and overall function.

Our study’s observed nanomechanical signatures likely reflect the dynamic interactions between CAR-T cells and the tumor microenvironment. T cells’ ability to sense and adapt to the stiffness and plasticity of their surroundings is fundamental to their efficacy in targeting cancer cells. The heterogeneously stiffer and more plastic tumor stroma in the combined therapy group may facilitate better infiltration and activation of CAR-T cells, enhancing their therapeutic potential.

Understanding immune cells’ mechanosensitive behaviors can provide deeper insights into their role in cancer therapy and help refine strategies for improving treatment outcomes. As we continue to explore these mechanical interactions, we aim to use this knowledge to develop more effective and targeted immunotherapies.

Finally, our findings underscore the therapeutic potential of combining cell therapy with LDRT, provided that it is tailored to the specific tumor type, and the adoptively transferred effector T cells are carefully matched with the radiation dosage. This innovative approach holds promise across a spectrum of preclinical models and has the potential for clinical trials, offering the unique advantage of inducing therapeutic effects without causing radiation-related tissue damage. Combining CAR-T cell therapy with RT, validated through successful preclinical studies, fuels high expectations for its forthcoming success in the clinic.

## Data Availability

The original contributions presented in the study are included in the article/**Supplementary Material**. Further inquiries can be directed to the corresponding authors.

## References

[1] Tsoumakidou M. The advent of immune stimulating CAFs in cancer. Nat Rev Cancer. (2023) 23:258–69. doi: 10.1038/s41568-023-00549-7 36807417

[2] Eskandari-Malayeri F Rezaei M. Immune checkpoint inhibitors as mediators for immunosuppression by cancer-associated fibroblasts: A comprehensive review. Front Immunol. (2022) 13:996145. doi: 10.3389/fimmu.2022.996145 36275750 PMC9581325

[3] Labani-Motlagh A Ashja-Mahdavi M Loskog A. The tumor microenvironment: A milieu hindering and obstructing antitumor immune responses. Front Immunol. (2020) 11:940. doi: 10.3389/fimmu.2020.00940 32499786 PMC7243284

[4] Jarnicki AG Lysaght J Todryk S Mills KH. Suppression of antitumor immunity by IL-10 and TGF-beta-producing T cells infiltrating the growing tumor: influence of tumor environment on the induction of CD4+ and CD8+ regulatory T cells. J Immunol. (2006) 177:896–904. doi: 10.4049/jimmunol.177.2.896 16818744

[5] Mariathasan S Turley SJ Nickles D Castiglioni A Yuen K Wang Y. TGFbeta attenuates tumour response to PD-L1 blockade by contributing to exclusion of T cells. Nature. (2018) 554:544–8. doi: 10.1038/nature25501 PMC6028240 29443960

[6] Menon H Chen D Ramapriyan R Verma V Barsoumian HB Cushman TR. Influence of low-dose radiation on abscopal responses in patients receiving high-dose radiation and immunotherapy. J Immunother Cancer. (2019) 7:237. doi: 10.1186/s40425-019-0718-6 31484556 PMC6727581

[7] Menon H Ramapriyan R Cushman TR Verma V Kim HH Schoenhals JE. Role of radiation therapy in modulation of the tumor stroma and microenvironment. Front Immunol. (2019) 10:193. doi: 10.3389/fimmu.2019.00193 30828330 PMC6384252

[8] Pankova D Chen Y Terajima M Schliekelman MJ Baird BN Fahrenholtz M. Cancer-associated fibroblasts induce a collagen cross-link switch in tumor stroma. Mol Cancer Res. (2016) 14:287–95. doi: 10.1158/1541-7786.MCR-15-0307 PMC4794404 26631572

[9] Nissen NI Karsdal M Willumsen N. Collagens and Cancer associated fibroblasts in the reactive stroma and its relation to Cancer biology. J Exp Clin Cancer Res. (2019) 38:115. doi: 10.1186/s13046-019-1110-6 30841909 PMC6404286

[10] Meng XM Nikolic-Paterson DJ Lan HY. TGF-beta: the master regulator of fibrosis. Nat Rev Nephrol. (2016) 12:325–38. doi: 10.1038/nrneph.2016.48 27108839

[11] Cirri P Chiarugi P. Cancer associated fibroblasts: the dark side of the coin. Am J Cancer Res. (2011) 1:482–97. https://www.ncbi.nlm.nih.gov/pubmed/21984967. PMC3186047 21984967

[12] J W J JB L D S ARM Sh F S MJM. Abscopal effect following radiation therapy in cancer patients: A new look from the immunological point of view. J BioMed Phys Eng. (2020) 10:537–42. doi: 10.31661/jbpe.v0i0.1066 PMC7416099 32802801

[13] Barsoumian HB Ramapriyan R Younes AI Caetano MS Menon H Comeaux NI. Low-dose radiation treatment enhances systemic antitumor immune responses by overcoming the inhibitory stroma. J Immunother Cancer. (2020) 8. doi: 10.1136/jitc-2020-000537 PMC7592253 33106386

[14] Barsoumian HB Sezen D Menon H Younes AI Hu Y He K. High plus low dose radiation strategy in combination with TIGIT and PD1 blockade to promote systemic antitumor responses. Cancers (Basel). (2022) 14. doi: 10.3390/cancers14010221 PMC8750272 35008385

[15] Sezen D Patel RR Tang C Onstad M Nagarajan P Patel SP. Immunotherapy combined with high- and low-dose radiation to all sites leads to complete clearance of disease in a patient with metastatic vaginal melanoma. Gynecol Oncol. (2021) 161:645–52. doi: 10.1016/j.ygyno.2021.03.017 PMC13033395 33795130

[16] Barsoumian HB Hsu J Nanez S Hu Y Hsu EY Riad TS. The radScopal technique as an immune adjuvant to treat cancer. Immuno. (2023) 3:74–85. https://www.mdpi.com/2673-5601/3/1/6.

[17] Caetano MS Younes AI Barsoumian HB Quigley M Menon H Gao C. Triple therapy with merTK and PD1 inhibition plus radiotherapy promotes abscopal antitumor immune responses. Clin Cancer Res. (2019) 25:7576–84. doi: 10.1158/1078-0432.CCR-19-0795 PMC6911635 31540976

[18] Chen D Barsoumian HB Yang L Younes AI Verma V Hu Y. SHP-2 and PD-L1 inhibition combined with radiotherapy enhances systemic antitumor effects in an anti-PD-1-resistant model of non-small cell lung cancer. Cancer Immunol Res. (2020) 8:883–94. doi: 10.1158/2326-6066.CIR-19-0744 PMC10173258 32299915

[19] Niknam S Barsoumian HB Schoenhals JE Jackson HL Yanamandra N Caetano MS. Radiation followed by OX40 stimulation drives local and abscopal antitumor effects in an anti-PD1-resistant lung tumor model. Clin Cancer Res. (2018) 24:5735–43. doi: 10.1158/1078-0432.CCR-17-3279 PMC6239963 29784675

[20] Schoenhals JE Seyedin SN Tang C Cortez MA Niknam S Tsouko E. Preclinical rationale and clinical considerations for radiotherapy plus immunotherapy: going beyond local control. Cancer J. (2016) 22:130–7. doi: 10.1097/PPO.0000000000000181 27111909

[21] Schoenhals JE Skrepnik T Selek U Cortez MA Li A Welsh JW. Optimizing radiotherapy with immunotherapeutic approaches. Adv Exp Med Biol. (2017) 995:53–71. doi: 10.1007/978-3-319-53156-4_3 28321812

[22] Theelen W Chen D Verma V Hobbs BP Peulen HMU Aerts J. Pembrolizumab with or without radiotherapy for metastatic non-small-cell lung cancer: a pooled analysis of two randomised trials. Lancet Respir Med. (2021) 9:467–75. doi: 10.1016/S2213-2600(20)30391-X 33096027

[23] Welsh JW Tang C de Groot P Naing A Hess KR Heymach JV. Phase II trial of ipilimumab with stereotactic radiation therapy for metastatic disease: outcomes, toxicities, and low-dose radiation-related abscopal responses. Cancer Immunol Res. (2019) 7:1903–9. doi: 10.1158/2326-6066.CIR-18-0793 PMC6891218 31658994

[24] DeSelm C Palomba ML Yahalom J Hamieh M Eyquem J Rajasekhar VK. Low-dose radiation conditioning enables CAR-T cells to mitigate antigen escape. Mol Ther. (2018) 26:2542–52. doi: 10.1016/j.ymthe.2018.09.008 PMC6225039 30415658

[25] He K Barsoumian HB Bertolet G Verma V Leuschner C Koay EJ. Novel use of low-dose radiotherapy to modulate the tumor microenvironment of liver metastases. Front Immunol. (2021) 12:812210. doi: 10.3389/fimmu.2021.812210 34975924 PMC8714746

[26] Tomayko MM Reynolds CP. Determination of subcutaneous tumor size in athymic (nude) mice. Cancer Chemother Pharmacol. (1989) 24:148–54. doi: 10.1007/BF00300234 2544306

[27] Chen H Lau MC Wong MT Newell EW Poidinger M Chen J. Cytofkit: A bioconductor package for an integrated mass cytometry data analysis pipeline. PloS Comput Biol. (2016) 12:e1005112. doi: 10.1371/journal.pcbi.1005112 27662185 PMC5035035

[28] John E Sader IL Mulvaney P Lee R. Method for the calibration of atomic force microscope cantilevers. Rev Sci Instrum. (1995) 66:3789–98. doi: 10.1063/1.1145439

[29] Glentis A Oertle P Mariani P Chikina A El Marjou F Attieh Y. Cancer-associated fibroblasts induce metalloprotease-independent cancer cell invasion of the basement membrane. Nat Commun. (2017) 8:924. doi: 10.1038/s41467-017-00985-8 29030636 PMC5640679

[30] Plodinec M Loparic M Monnier CA Obermann EC Zanetti-Dallenbach R Oertle P. The nanomechanical signature of breast cancer. Nat Nanotechnol. (2012) 7:757–65. doi: 10.1038/nnano.2012.167 23085644

[31] Plodinec M Lim RY. Nanomechanical characterization of living mammary tissues by atomic force microscopy. Methods Mol Biol. (2015) 1293:231–46. doi: 10.1007/978-1-4939-2519-3_14 26040692

[32] Oliver WC Pharr GM. An improved technique for determining hardness and elastic modulus using load and displacement sensing indentation experiments. J Mater Res. (1992) 7:1564–83. doi: 10.1557/JMR.1992.1564

[33] Plodinec M Loparic M Suetterlin R Herrmann H Aebi U Schoenenberger CA. The nanomechanical properties of rat fibroblasts are modulated by interfering with the vimentin intermediate filament system. J Struct Biol. (2011) 174:476–84. doi: 10.1016/j.jsb.2011.03.011 21426942

[34] Nečas D Klapetek P. Gwyddion: an open-source software for SPM data analysis. centreurjphys. (2012) 10:181–8. doi: 10.2478/s11534-011-0096-2

[35] Hennessy M Wahba A Felix K Cabrera M Segura MG Kundra V. Bempegaldesleukin (BEMPEG; NKTR-214) efficacy as a single agent and in combination with checkpoint-inhibitor therapy in mouse models of osteosarcoma. Int J Cancer. (2021) 148:1928–37. doi: 10.1002/ijc.33382 PMC7984260 33152115

[36] Shen L Xiao Y Tian J Lu Z. Remodeling metabolic fitness: Strategies for improving the efficacy of chimeric antigen receptor T cell therapy. Cancer Lett. (2022) 529:139–52. doi: 10.1016/j.canlet.2022.01.006 35007698

[37] Mehta PH Fiorenza S Koldej RM Jaworowski A Ritchie DS Quinn KM. T cell fitness and autologous CAR-T cell therapy in haematologic Malignancy. Front Immunol. (2021) 12:780442. doi: 10.3389/fimmu.2021.780442 34899742 PMC8658247

[38] Brock SE Rendon BE Yaddanapudi K Mitchell RA. Negative regulation of AMP-activated protein kinase (AMPK) activity by macrophage migration inhibitory factor (MIF) family members in non-small cell lung carcinomas. J Biol Chem. (2012) 287:37917–25. doi: 10.1074/jbc.M112.378299 PMC3488063 22988252

[39] Patel RR He K Barsoumian HB Chang JY Tang C Verma V. High-dose irradiation in combination with non-ablative low-dose radiation to treat metastatic disease after progression on immunotherapy: Results of a phase II trial. Radiother Oncol. (2021) 162:60–7. doi: 10.1016/j.radonc.2021.06.037 PMC11905861 34237343

[40] Felgentreff K Schuetz C Baumann U Klemann C Viemann D Ursu S. Differential DNA damage response of peripheral blood lymphocyte populations. Front Immunol. (2021) 12:739675. doi: 10.3389/fimmu.2021.739675 34594342 PMC8478158

[41] Tharp KM Park S Timblin GA Richards AL Berg JA Twells NM. The microenvironment dictates glycocalyx construction and immune surveillance. Res Sq. (2023). doi: 10.21203/rs.3.rs-3164966/v1

[42] Mittelheisser V Gensbittel V Bonati L Li W Tang L Goetz JG. Evidence and therapeutic implications of biomechanically regulated immunosurveillance in cancer and other diseases. Nat Nanotechnol. (2024) 19:281–97. doi: 10.1038/s41565-023-01535-8 38286876

[43] Jiang Y Zhang H Wang J Liu Y Luo T Hua H. Targeting extracellular matrix stiffness and mechanotransducers to improve cancer therapy. J Hematol Oncol. (2022) 15:34. doi: 10.1186/s13045-022-01252-0 35331296 PMC8943941

[44] Walker JM Rolig AS Charych DH Hoch U Kasiewicz MJ Rose DC. NKTR-214 immunotherapy synergizes with radiotherapy to stimulate systemic CD8(+) T cell responses capable of curing multi-focal cancer. J Immunother Cancer. (2020) 8. doi: 10.1136/jitc-2019-000464 PMC7252958 32457127

[45] Nanjireddy PM Olejniczak SH Buxbaum NP. Targeting of chimeric antigen receptor T cell metabolism to improve therapeutic outcomes. Front Immunol. (2023) 14:1121565. doi: 10.3389/fimmu.2023.1121565 36999013 PMC10043186

