# Supplementary figures and images for: Enhanced tumor control and survival in preclinical models with adoptive cell therapy preceded by low-dose radiotherapy
Source: Front Oncol. 2024 Oct 9;14:1407143. doi: 10.3389/fonc.2024.1407143 (PMC11496962; doi:10.3389/fonc.2024.1407143)

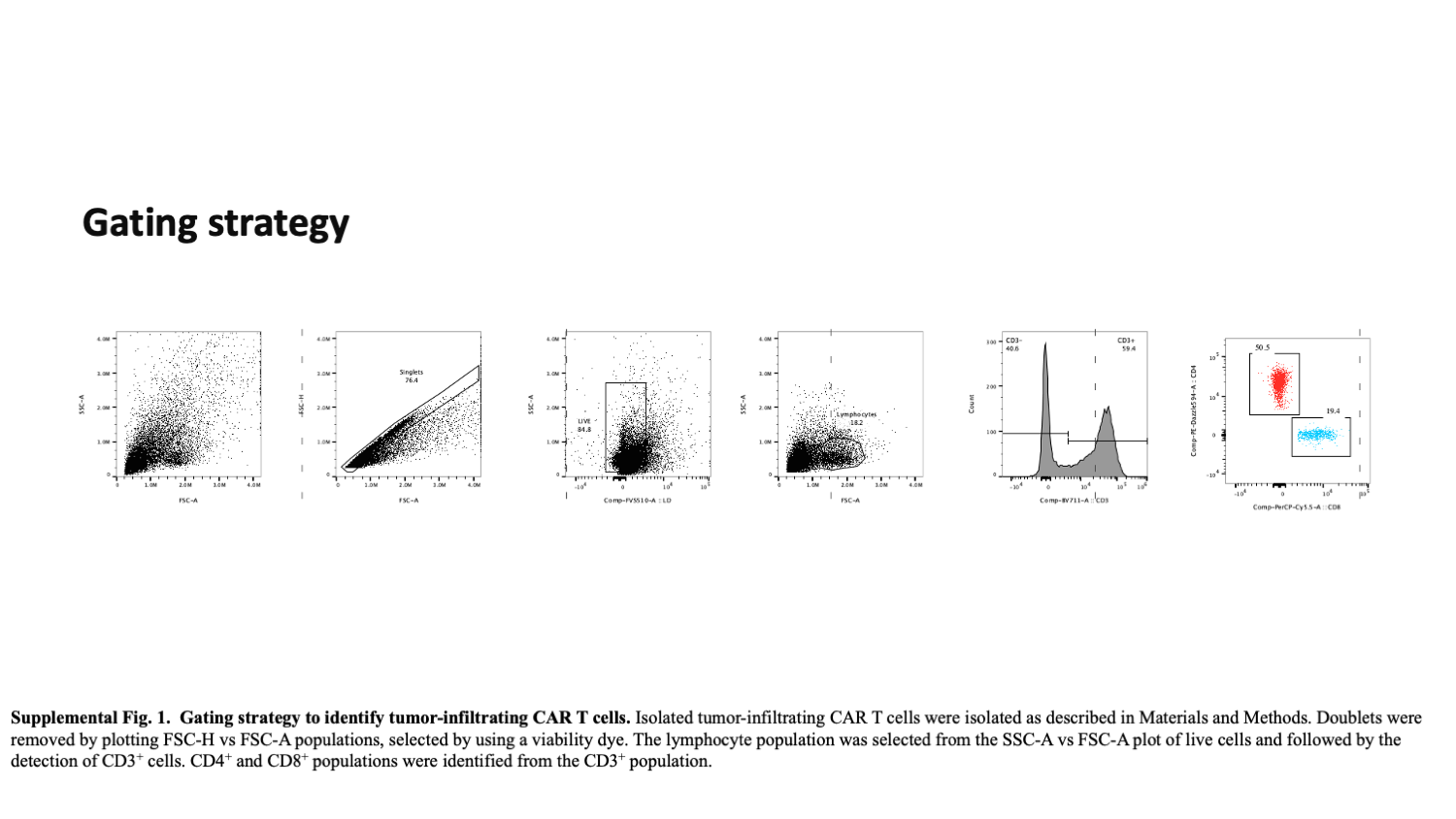

Supplement: Supplementary file 1 [file Image1.tiff]

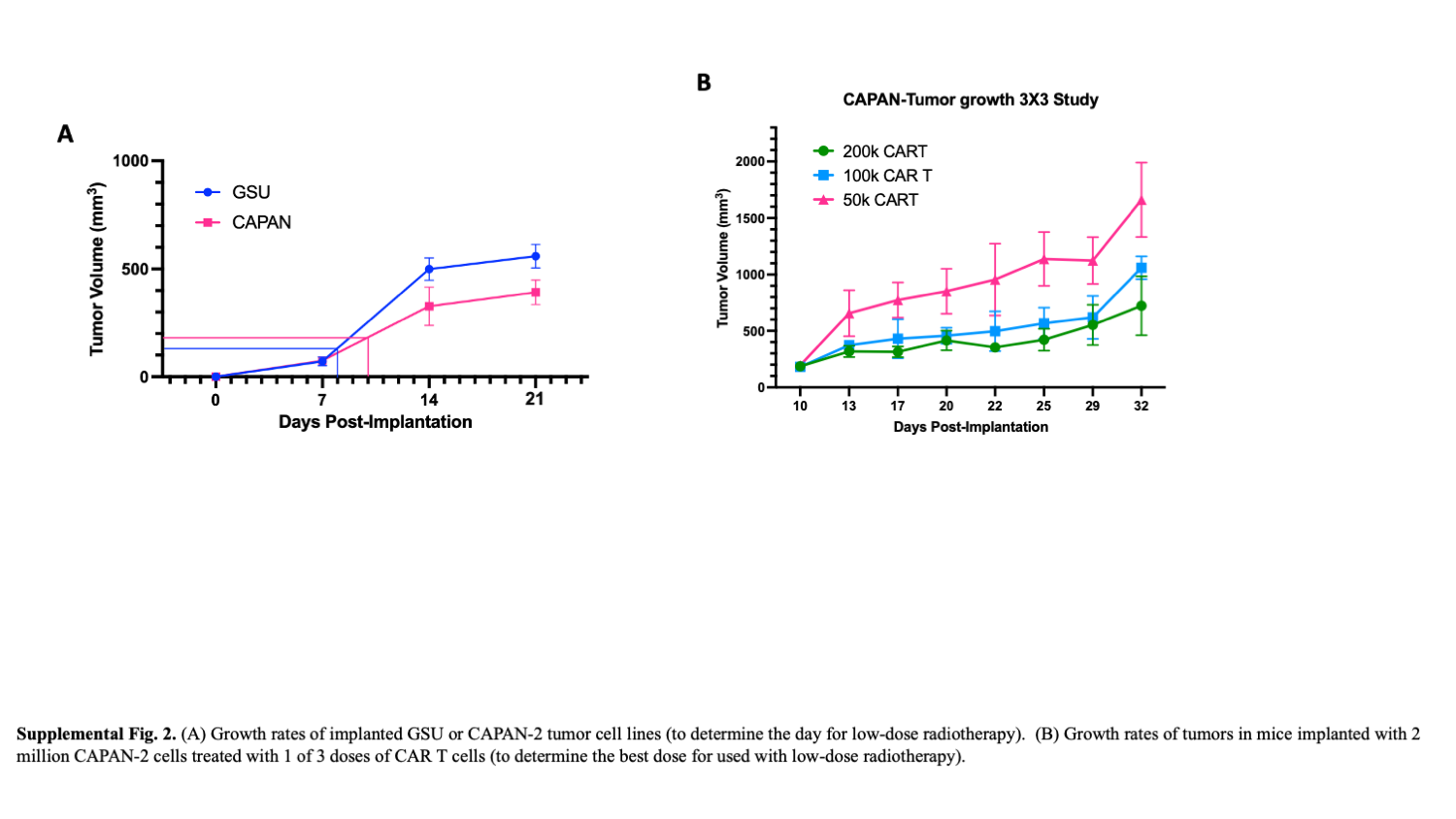

Supplement: Supplementary file 2 [file Image2.tiff]

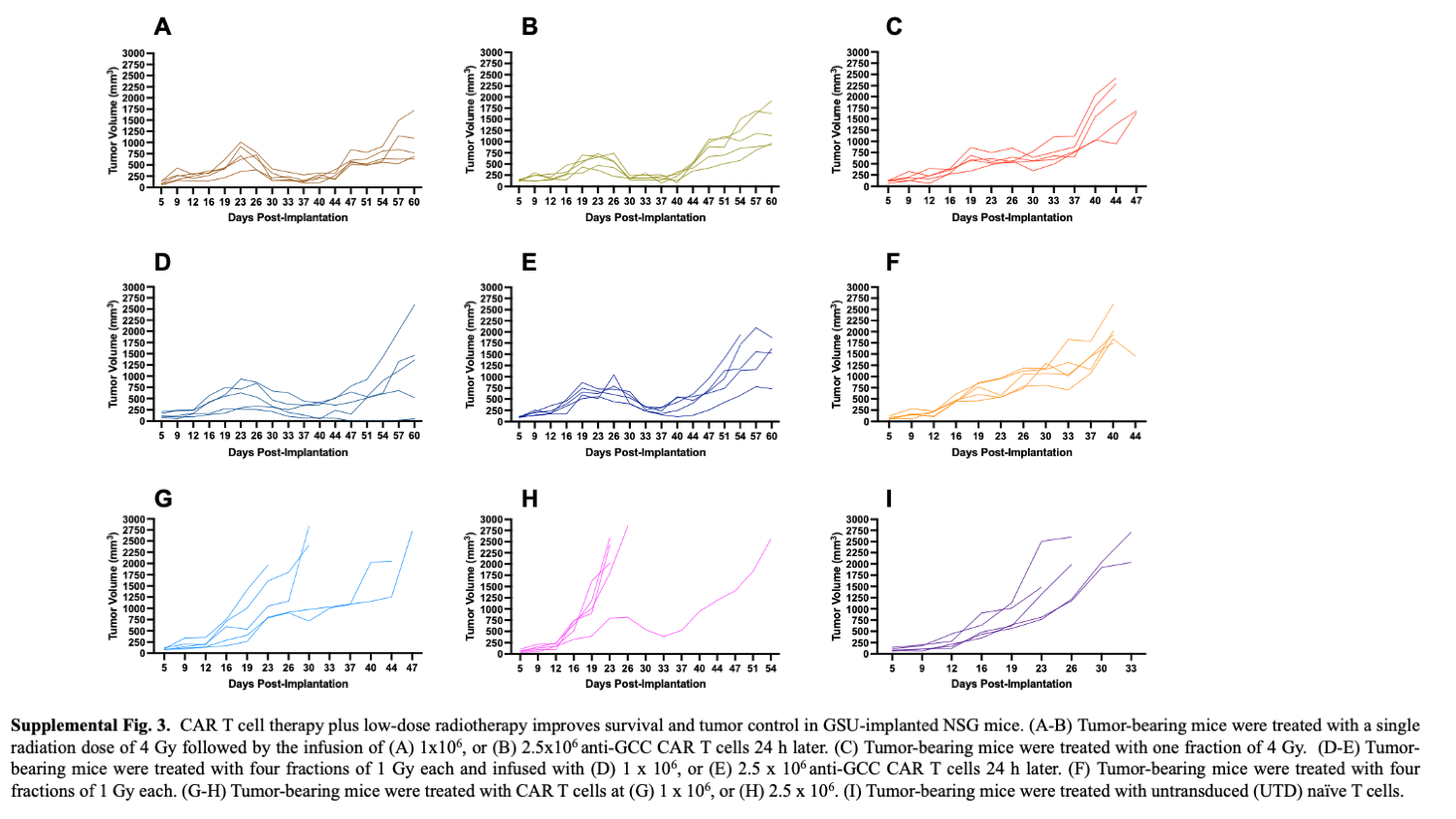

Supplement: Supplementary file 3 [file Image3.tiff]

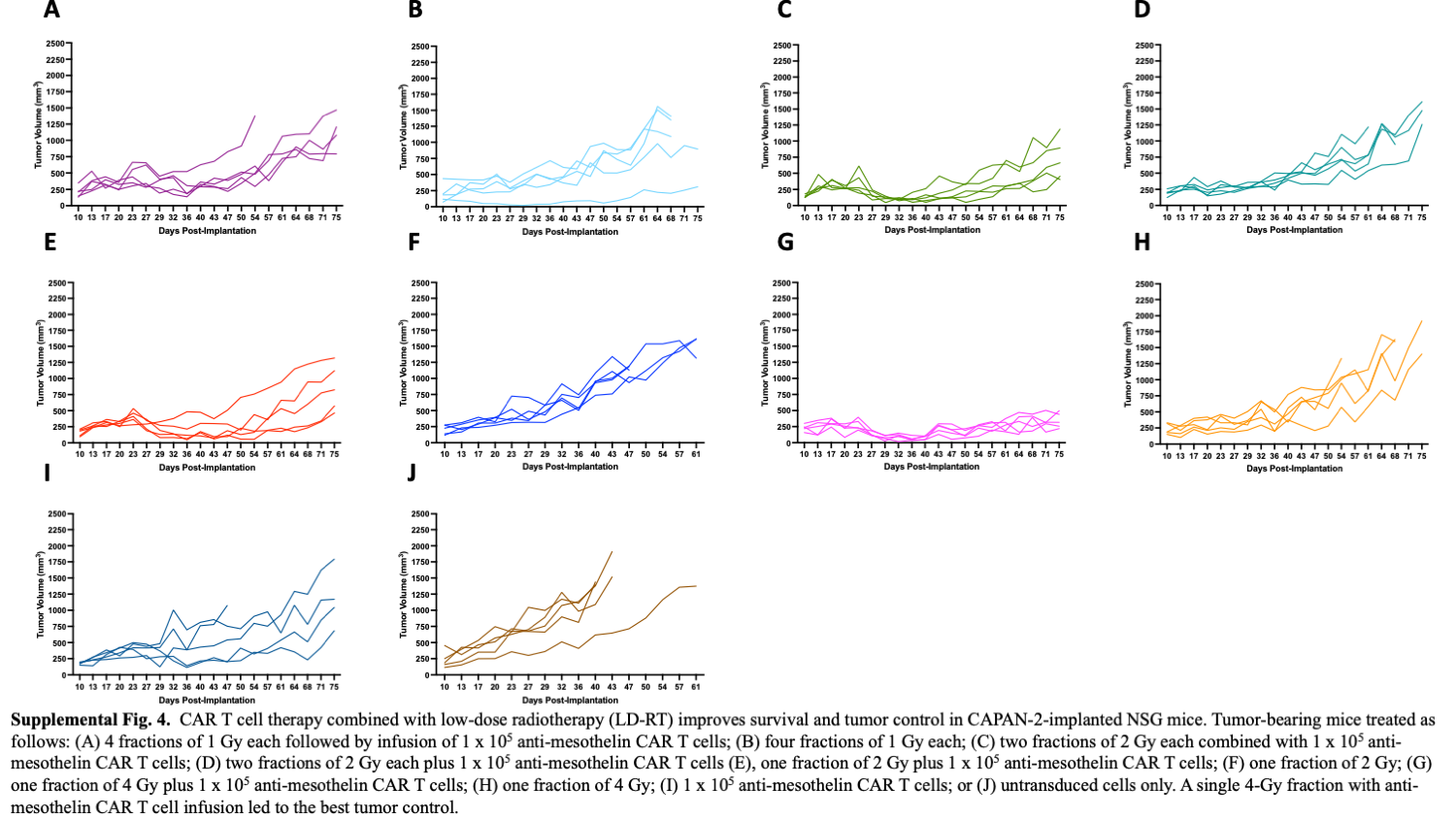

Supplement: Supplementary file 4 [file Image4.tiff]

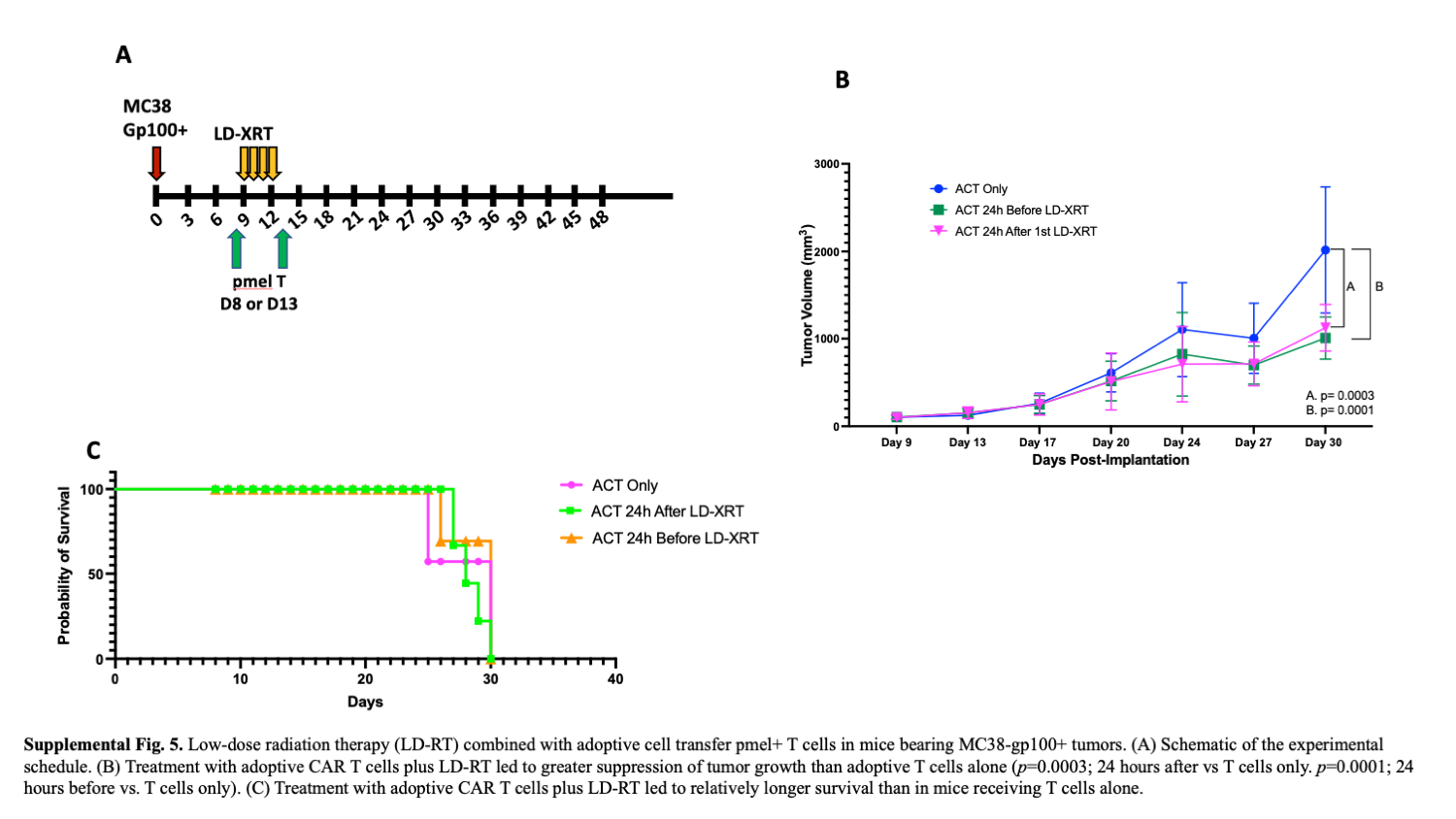

Supplement: Supplementary file 5 [file Image5.tiff]

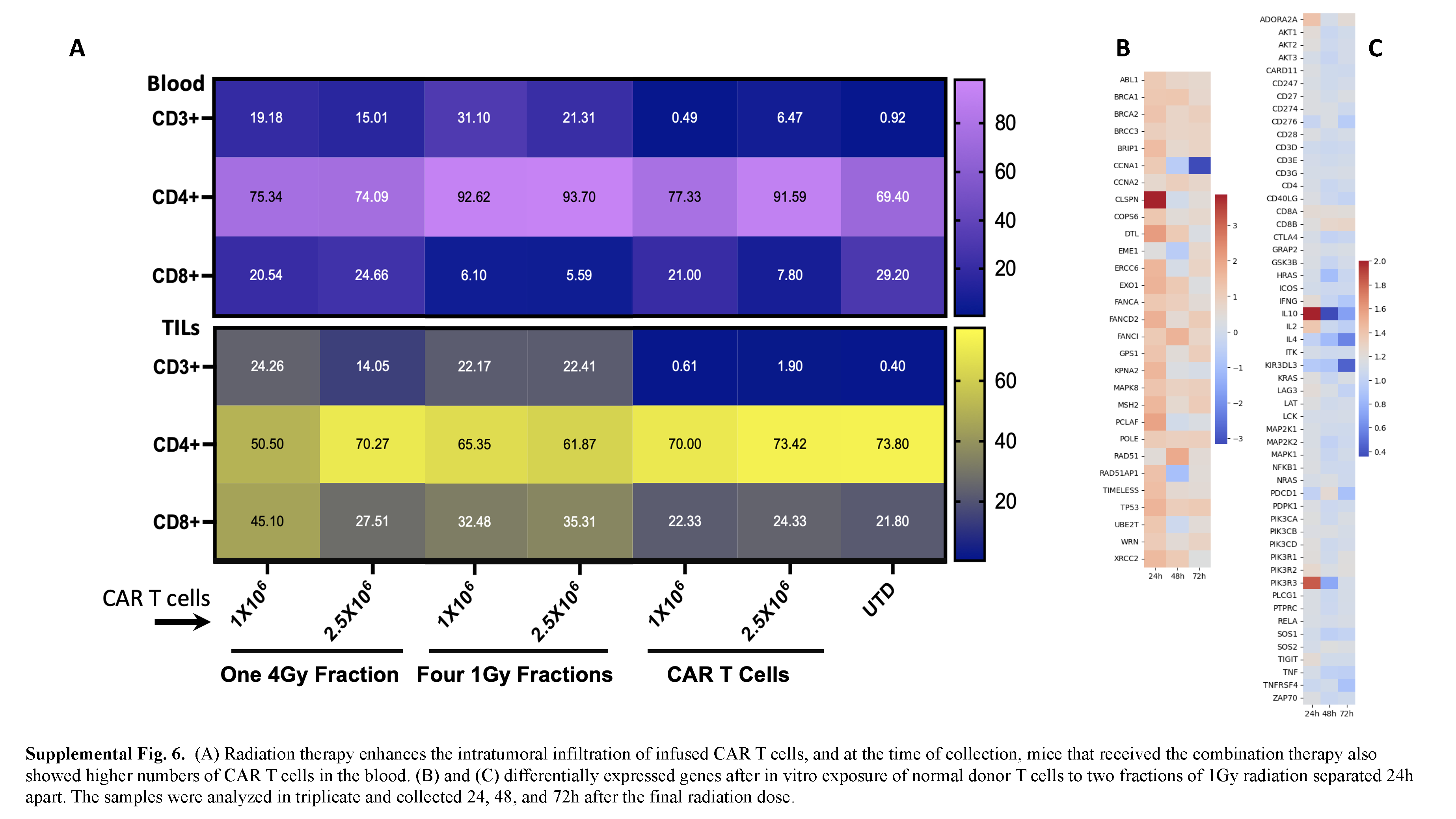

Supplement: Supplementary file 6 [file Image6.tiff]

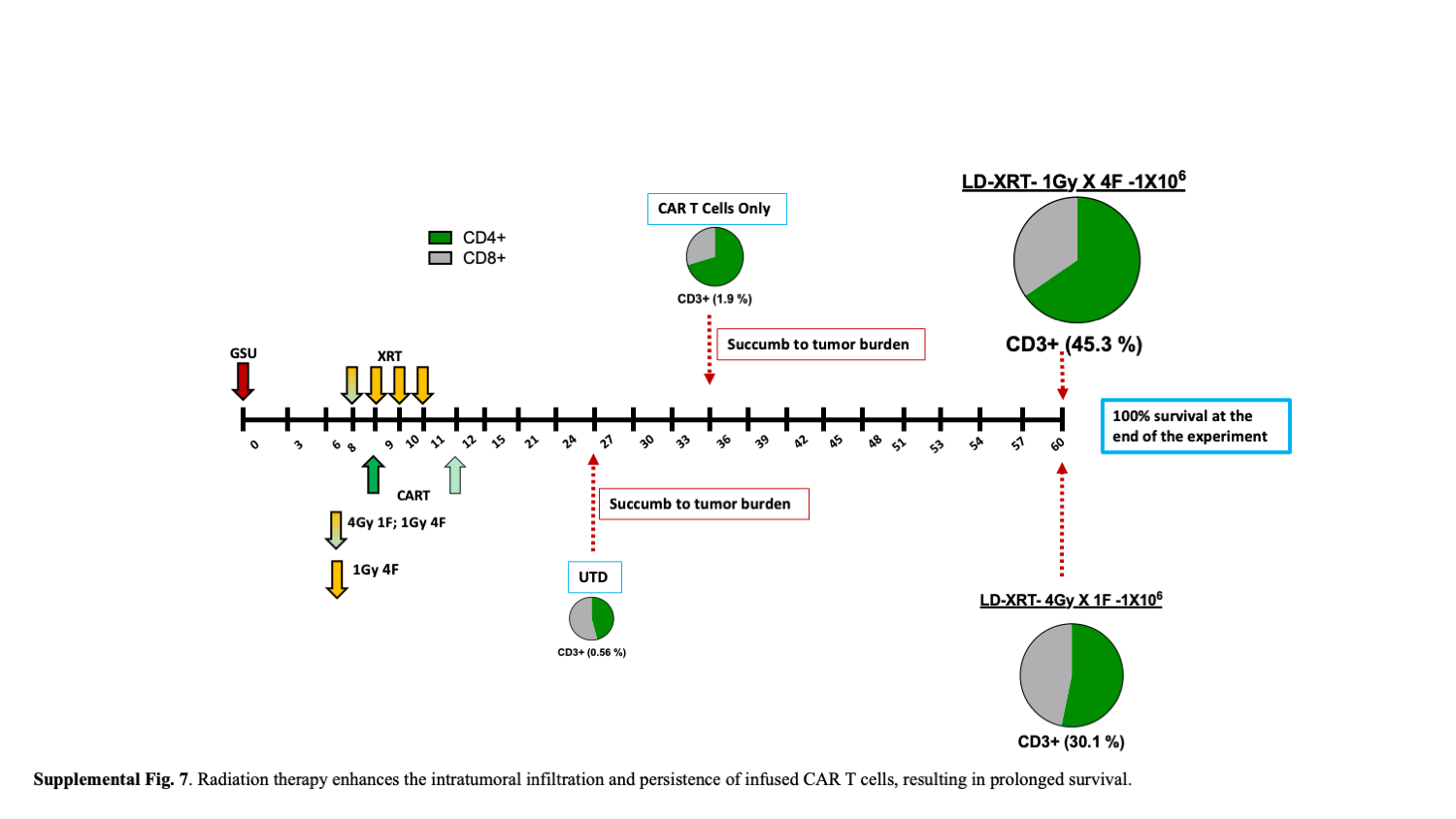

Supplement: Supplementary file 7 [file Image7.tiff]

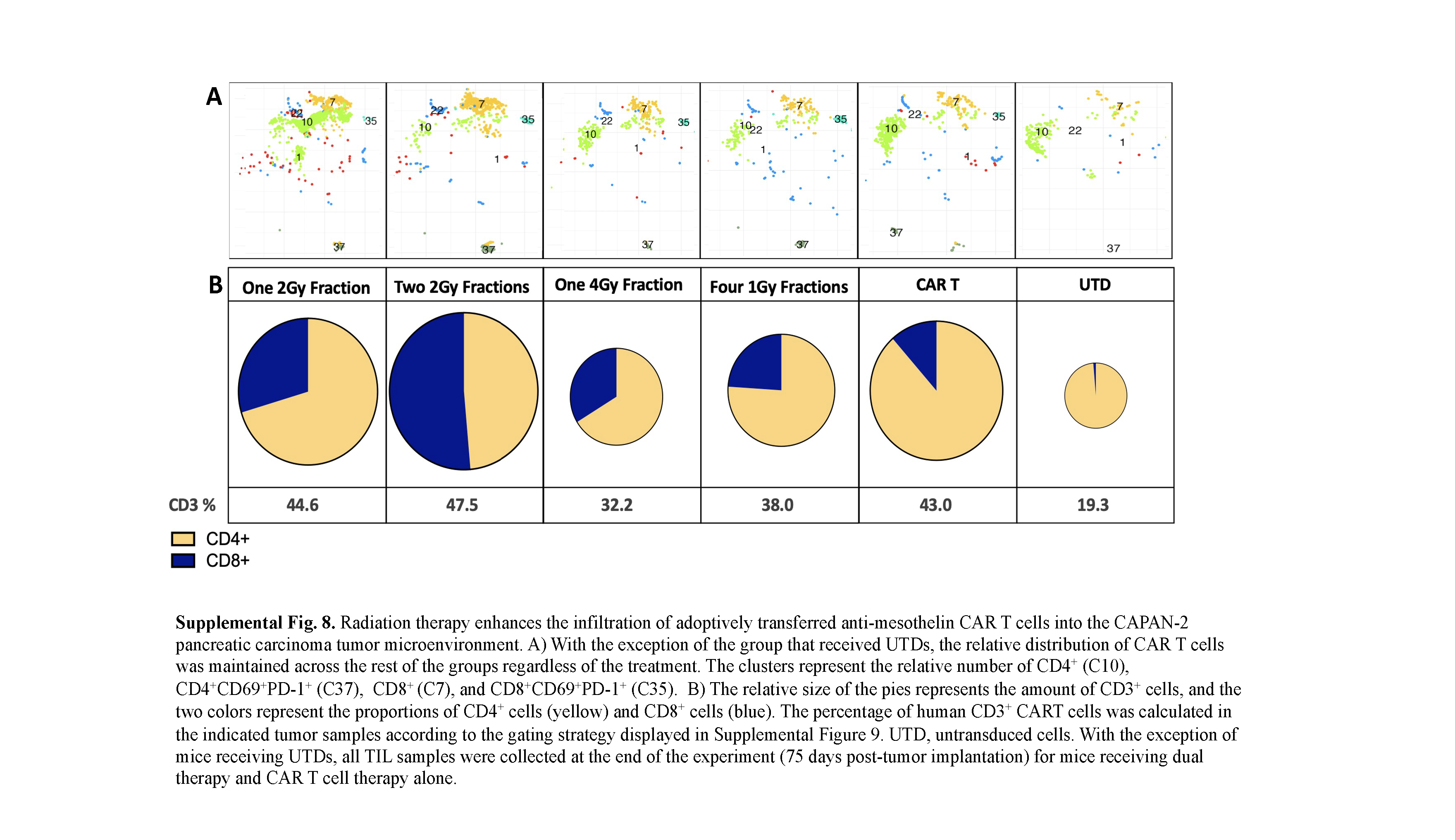

Supplement: Supplementary file 8 [file Image8.tiff]

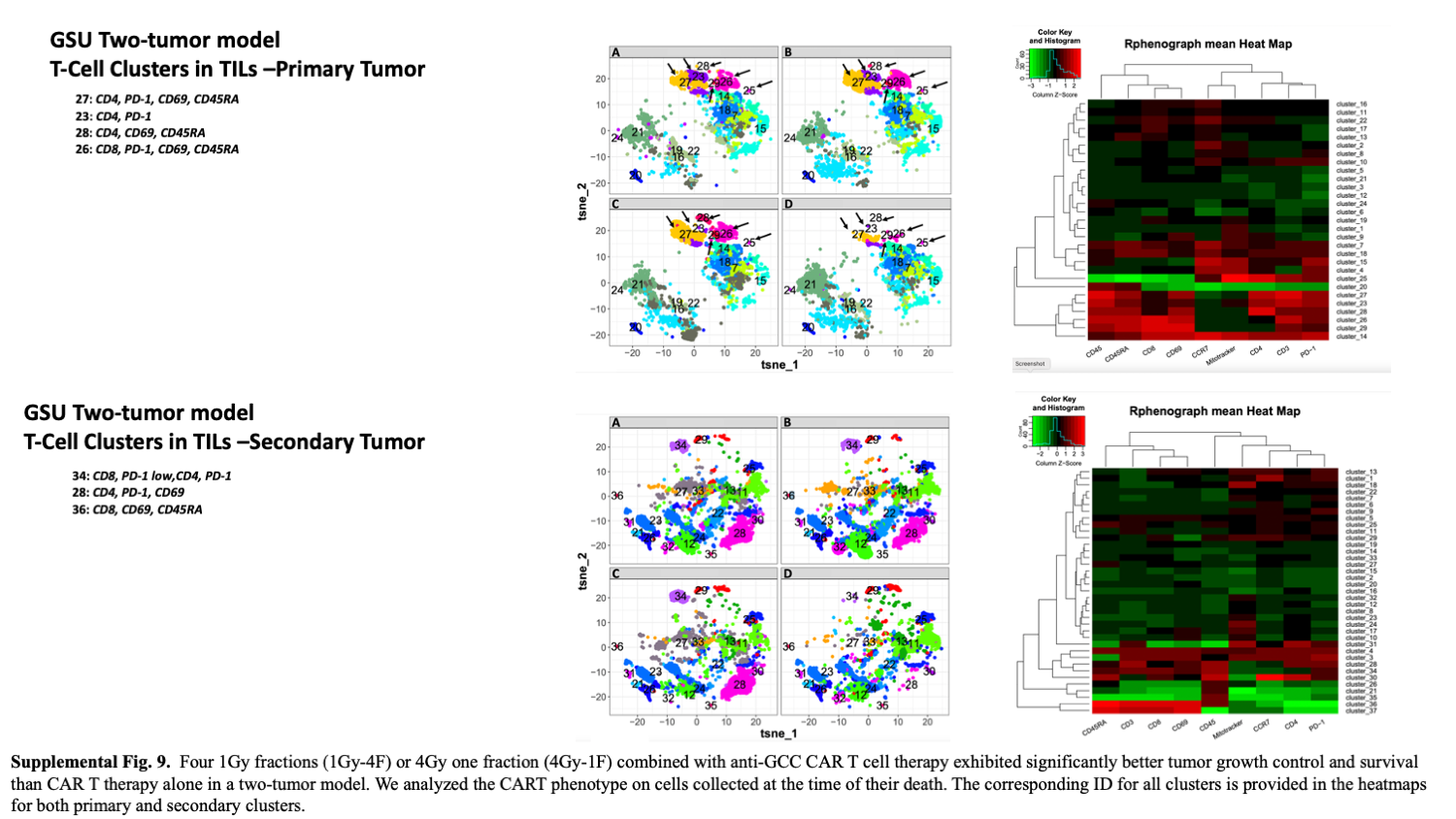

Supplement: Supplementary file 9 [file Image9.tiff]
